# Definitions of Severity in Treatment Seeking Studies of Febrile Illness in Children in Low and Middle Income Countries: A Scoping Review

**DOI:** 10.3389/ijph.2021.634000

**Published:** 2021-08-30

**Authors:** Nina C. Brunner, Phyllis Awor, Manuel W. Hetzel

**Affiliations:** ^1^ Department of Epidemiology and Public Health, Swiss Tropical and Public Health Institute, Basel, Switzerland; ^2^ University of Basel, Basel, Switzerland; ^3^ Department of Community Health and Behavioural Sciences, Makerere University School of Public Health, Kampala, Uganda

**Keywords:** fever, severity, child, developing countries, treatment seeking, health behavior

## Abstract

**Objectives:** Understanding treatment seeking for severe febrile illness (SFI) is methodologically challenging. In this scoping review, we investigate definitions of severe febrile illness in treatment seeking studies on children under 5 years of age in low and middle income countries. We analyze the association of SFI definitions with different concepts of treatment seeking and identify related research gaps.

**Methods:** We searched Pubmed, Scopus and WHOLIS, and screened references of included publications for eligibility.

**Results:** Definitions of SFI had either a biomedical perspective (predominantly in quantitative studies) or a caregiver perspective (predominantly in qualitative studies). In quantitative analyses of treatment seeking, severity was more often conceptualized as a determinant rather than an outcome of a treatment seeking process. The majority of quantitative analyses only included surviving children or did not explicitly mention dead children.

**Conclusion:** Different research questions lead to diverse definitions and concepts of severity and treatment seeking outcomes, which limits the comparability of the available evidence. Systematic exclusion of dead children is likely to bias inferences on the association of treatment seeking and health outcomes of children with SFI in low and middle income countries.

## Introduction

In 2019, low and middle income countries (LMICs) accounted for 99% of the 5.2 million deaths in children under 5 years of age worldwide [1]. Despite important reductions, infectious causes such as pneumonia, diarrhea, and malaria continue to significantly contribute to child mortality [2]. Early and appropriate treatment can considerably reduce deaths from infectious causes, preventing the progression from mild to severe disease and death [3]. Prompt and appropriate treatment seeking therefore plays an important role in achieving the Sustainable Development Goal 3.2 target of reducing under five mortality to 25 per 1,000 live births [4].

Reciprocally, severity of illness influences health seeking behavior [5]. The existing scientific literature includes several reviews of treatment seeking for childhood illnesses which take into consideration severity of illness as a determinant of the care seeking process [6-10]. Most of these reviews have focused on common childhood illnesses such as malaria, diarrhea, and/or pneumonia. However, due to the clinical overlap of malaria with pneumonia and other infectious diseases [11-15] and the high case fatality ratio among admitted patients with febrile illness, etiology research has suggested a syndromic approach for research on severe febrile illness (SFI) [16]. This approach is particularly relevant to treatment seeking research because caregivers recognize and classify their child’s illness based on observed signs and symptoms. Synthesizing the evidence on treatment seeking for SFI in children from a syndromic perspective can provide valuable insights into the mechanisms connecting illness presentation, care seeking, and health outcomes. Analyzing the underlying methodological approaches of such studies will help to understand the validity and comparability of the available evidence.

The concept of SFI has been referred to by several studies. Even though Roddy et al. [17] describe SFI as “a commonly documented and globally recognized cause of morbidity and mortality,” definitions of SFI in the scientific literature vary. While some authors have focused on clinical diagnosis [13, 17] or causal agents [16], others defined SFI as fever with danger signs according to the Integrated Management of Childhood Illness (IMCI) guidelines [18]. Health behavior theories have also pointed out the importance of perceived severity for health-related behavior such as treatment seeking [19‐21].

Considering the importance of severity for treatment seeking and health outcomes in children under five and the ambiguity of SFI definitions in the existing scientific literature, this scoping review aims at investigating the definitions of SFI and concepts of severity in studies on treatment seeking for febrile illness in children under 5. Additionally, we assessed potential sources of bias in the study designs of treatment seeking studies for SFI to identify research gaps.

## Methods

For this scoping review, we followed the approach suggested by Arksey and O'Malley (2005) [22] with the following key stages:

### Stage 1: Identifying Research Question

The purpose of this review is to explore definitions of SFI, concepts of severity, and research methods used in studies on treatment seeking for SFI in children under five in LMICs. SFI was defined and categorized iteratively throughout the screening and coding process. Treatment seeking was defined as seeking help outside of home from any provider even if the treatment prescribed by the provider was used for home management. LMICs were defined according to the World Bank classification of countries by income [23].

### Stage 2: Identifying Relevant Studies

We searched PubMed, Scopus and WHOLIS (last update on April 29, 2020) without restrictions of the publication date. Studies published in English or French were included. Malaria and pneumonia were used synonymously with fever based on previous research showing a high sensitivity of fever for malaria and pneumonia diagnosis [24‐27]. We adapted the search filter for pediatric studies proposed by Leclercq, et al. [28] and used the search filter for LMICs suggested by The Cochrane Collaboration [29]. No search filter was used for severe illness to allow for an iterative exploration of SFI definitions. The search terms and filters used are compiled in Supplementary Table S1 (Supplementary Material).

### Stage 3: Study Selection

All citations were imported into Zotero 5 (Corporation for Digital Scholarship, Vienna, VA, United States) and duplicates removed manually. Studies were included if all inclusion and exclusion criteria were met (Table 1). One researcher screened titles, abstracts and full text of the citations. Studies for which no abstract was available were put forward to full text screening. Articles were excluded from further analysis if they could not be obtained as full text through online databases and library searches at the University of Basel. Reference lists of included studies were screened to identify additional references.

**TABLE 1:** Inclusion and exclusion criteria of studies (Definitions of severity in treatment seeking studies of febrile illness in children in low and middle income countries: a scoping review, Switzerland, 2021).

**Inclusion criteria**
General • Study describes treatment seeking from a health provider outside home • Published online articles in English or French • Febrile illness is classified as severe by study authors, perceived as severe by study participants, or severity was investigated as a determinant of treatment seeking^a^
Quantitative studies • At least 80% of study participants had fever, malaria or pneumonia, or results for study participants with fever, malaria or pneumonia are reported separately • At least 80% of study participants are children under 5 or results for children under 5 are reported separately from other age groups
Qualitative studies • Study participants are caregivers of children under 5 • Study participants associate the discussed illness with fever, malaria or pneumonia
**Exclusion criteria**
• Studies exclusively focusing on newborns (0–2 months) • Literature reviews • Studies using modeled data to describe treatment seeking

a Synonyms for severe: serious, fatal, deadly, life-threatening, complicated, dangerous, and related nouns.

### Stage 4: Extracting and Analyzing the Data

Relevant information was extracted from selected articles using MaxQDA Plus 2018 (VERBI GmbH, Berlin, Germany). Categories of study characteristics were defined a priori and provided the coding framework for data extraction. For some categories, codes were defined a priori (Table 2). Codes emerging from the included manuscripts are shown in Table 3 (year of publication, study location, disease, inclusion criteria, treatment seeking outcome, concept of severity) and Table 4 (definitions of SFI) (see Results). Definitions of SFI were extracted from all included articles. For quantitative analyses, we iteratively explored concepts of severity, extracted treatment seeking outcomes, as well as inclusion and exclusion criteria to assess bias. Treatment seeking outcomes were categorized into: 1) treatment seeking proportion 2) referral adherence, 3) sources of treatment, and 4) delays in treatment seeking. Only treatment seeking outcomes in relation to SFI were coded.

**TABLE 2:** Category and code definitions for data extraction from included publications (Definitions of severity in treatment seeking studies of febrile illness in children in low and middle income countries: a scoping review, Switzerland, 2021).

Category	Codes defined a priori	Definition
Year of publication	—	Year in which the study was published
Study location	—	Country in which the study took place
Disease	—	Disease providing the context for the study
Method for data analysis	Quantitative	Treatment seeking for SFI was analyzed using quantitative methods
	Qualitative	Treatment seeking for SFI was analyzed using qualitative methods
	Mixed methods	Treatment seeking for SFI was analyzed using quantitative and qualitative methods
Participant enrolment	Community	Participants are enrolled from their location of home
	Health care provider	Participants are enrolled at a health care provider upon attendance or were identified as eligible participants through the screening of patient register entries
	Community + health care provider	Participants are enrolled from their location of home and at health care providers
Sample size (quantitative analyses only)	All	Total number of illness episodes included in the quantitative treatment seeking research component
	SFI	Number of SFI episodes included in the quantitative treatment seeking research component
Definition of SFI	—	Descriptions of SFI. SFI is any febrile condition that is described as severe, serious, complicated, dangerous, fatal, deadly, or life threatening. The definition of severity may be pre-defined or emerge from the findings of the study
Concepts of severity in quantitative analyses	—	Severity may play different roles in quantitative analyses of treatment seeking (e.g., covariate, inclusion criteria, etc.). The exploration of this concept is iterative and inductive
Inclusion criteria (quantitative analyses only)	—	Inclusion criteria for the enrolment of participants
Exclusion criteria (quantitative analyses only)	—	Exclusion criteria for the enrolment of participants
Treatment seeking outcomes (quantitative analyses only)	Treatment seeking proportion	The study outcome classifies treatment seeking actions for SFI into seeking care from outside home and not seeking care from outside of home. For studies enrolling participants from health care providers, the treatment seeking proportion distinguishes between children seeking care from another provider and children directly coming from home before attending the enrolling provider
	Referral adherence	Dichotomous variable that distinguishes between adhering and not adhering to referral advice for SFI.
	Other dichotomous outcome	Other dichotomous treatment seeking outcomes for SFI, e.g., seeking care from appropriate provider vs. seeking care from inappropriate provider
	Sources of treatment	Treatment seeking outcomes are classified into different sources of treatment for SFI.
	Delays	Delays can either be defined as a threshold after which treatment seeking is considered to be delayed, or the treatment seeking outcome is reported as the mean or median time until treatment

**TABLE 3:** Studies meeting the inclusion criteria of the scoping review (Definitions of severity in treatment seeking studies of febrile illness in children in low and middle income countries: a scoping review, Switzerland, 2021).

First author and year of publication	Country	Context	Disease	Method	Enrolment	SFI definition^a^	Inclusion criteria^b^	Treatment seeking outcome indicator^c^	Concept of severity^d^
Adedire (2015)	Nigeria	Rural	Fever	Mixed methods	Provider	6, 8	1, 2, 8, 9	2	2
Agyepong (1994)	Ghana	Rural + Urban	Malaria	Qualitative	Community	6	NA	NA	NA
Ahorlu (2005)	Ghana	Rural	Malaria	Mixed methods	Community	5	1	1, 4, 5	1
Ajayi et al [30]	Burkina Faso, Nigeria, Uganda	Rural	Malaria	Quantitative	Provider	2	1, 6, 7, 8, 9	1, 3, 4, 5	6
Akogun et al. [31]	Nigeria	Rural	Malaria	Qualitative	Community	5	NA	NA	NA
Amuyunzu (2006)	Keya	Urban	Pneumonia/ARI	Qualitative	Community	8	NA	NA	NA
Anaba et al. [32]	Nigeria	Unknown	Pneumonia/ARI	Quantitative	Community	6	1, 8	2	2
Awasthi (2015)	India	Rural	Pneumonia/ARI	Qualitative	Provider	2, 6	NA	NA	NA
Bantie (2019)	Ethiopia	Rural + Urban	Pneumonia/ARI	Quantitative	Provider	1	1, 4, 8, 9	5	2
Baume et al. [33]	Zambia	Rural + Urban	Malaria	Mixed methods	Community	8	1, 2, 5, 8	1, 4	1
Beiersmann et al. [34]	Burkina Faso	Rural	Malaria	Mixed methods	Community	3, 5	1, 3, 6, 7	1, 4	4
Bruce (2014)	Guatemala	Rural	Pneumonia/ARI	Quantitative	Community	2	1, 4, 5, 6, 8	2, 5	3
Burton (2011)	Kenya	Rural	Fever	Quantitative	Community	2	1, 2, 4, 5, 8	2	2
Chibwana et al. [35]	Malawi	Rural	Malaria	Qualitative	Community	8	NA	NA	NA
Comoro (2003)	Tanzania	Rural + Urban	Malaria	Qualitative	Provider	5	NA	NA	NA
Dada (2007)	Nigeria	Urban	Malaria	Quantitative	Provider	4	1, 2, 3, 8, 9	5	5
de Savigny et al. [36]	Tanzania	Rural	Malaria	Mixed methods	Community	3, 5	1, 3, 5, 6, 7	1, 4	4
Desmond (2013)	Malawi	Urban	Meningitis	Qualitative	Community + Provider	8	NA	NA	NA
Deutscher (2012)	Kenya, Guatemala, Thailand	Rural + Urban	Pneumonia/ARI	Quantitative	Community	2	1, 4, 5, 6, 8	2	3
Dillip et al. [37]	Tanzania	Rural + Urban	Malaria	Mixed methods	Community	5	1, 2, 5, 8	2, 5	1, 3
Dillip et al. [38]	Tanzania	Rural + Urban	Malaria	Mixed methods	Community	5	1, 5, 6, 8	2, 5	3
Do et al. [39]	Madagascar, Mali, Nigeria	Rural + Urban	Malaria	Quantitative	Community	6	1, 2, 5, 8	2	2
Druetz et al. [40]	Burkina Faso	Rural + Urban	Malaria	Quantitative	Community	2	1, 5, 8	2	2
Elimian (2020)	Nigeria	Urban	Pneumonia/ARI, Malaria	Qualitative	Provider	6	NA	NA	NA
Ellis et al. [18]	Mali	Rural	Malaria	Mixed methods	Community	2	1, 2, 5, 6, 7, 8	1, 4	3
Escribano-Ferrer et al.. [41]	Ghana	Rural	Malaria	Quantitative	Community	4	1, 2, 4, 5, 8	5	3
Ewing (2015)	Malawi	Rural + Urban	Malaria	Qualitative	Community	5	NA	NA	NA
Ferdous (2014)	Bangladesh	Rural	Pneumonia/ARI	Qualitative	Provider	8	NA	NA	NA
Ferdous (2018)	Bangladesh	Rural	Pneumonia/ARI	Quantitative	Community	3	1, 6, 7	1, 5	5
Foster and Vildendrer [42]	Tanzania	Rural + Urban	Malaria	Qualitative	Community	5	NA	NA	NA
Hausmann-Muela (2000)	Tanzania	Urban	Malaria	Mixed methods	Community	5	1	4	3
Hildenwall (2008)	Uganda	Rural	Pneumonia/ARI, Malaria	Mixed methods	Community	3	1, 2, 5, 6, 7	1, 4, 5	4
Hildenwall (2009a)	Uganda	Urban	Pneumonia/ARI	Quantitative	Provider	2	1, 4, 6, 8, 9	1, 5	3
Hildenwall (2009b)	Uganda	Urban	Pneumonia/ARI	Quantitative	Provider	2	1, 4, 6, 7, 8, 10	1, 4	5
Houéto (2007)	Benin	Rural	Malaria	Qualitative	Community	7, 8	NA	NA	NA
Kaatano (2006)	Tanzania	Rural	Malaria	Quantitative	Community	4	1, 2, 5, 8	1, 4	1
Kamat [43]	Tanzania	Urban	Malaria	Qualitative	Provider	5, 6, 7	NA	NA	NA
Kamat (2008)	Tanzania	Urban	Malaria	Mixed methods	Community + Provider	1, 5	1, 9	4	3
Kaona (2005)	Zambia	Rural	Malaria	Qualitative	Community + Provider	5	NA	NA	NA
Kapoor (1990)	India	Rural	Pneumonia/ARI	Quantitative	Community	4	1, 8	1	1
Kassam et al. [44]	Uganda	Rural + Urban	Malaria	Quantitative	Community	6	1, 2, 5, 8	1	1
Kassam et al. [45]	Uganda	Rural + Urban	Malaria	Qualitative	Community	3	NA	NA	NA
Kassile et al. [46]	Tanzania	Rural + Urban	Malaria	Quantitative	Community	6	1, 2, 5, 8	5	2
Kerai (2019)	Pakistan	Urban	Pneumonia/ARI	Quantitative	Community	2	1, 5, 8	1, 4	1, 2
Kosai (2015)	Phillipines	Rural + Urban	Pneumonia/ARI	Quantitative	Community	2	1, 4, 7, 8	2	1, 3
Källander (2008)	Uganda	Rural	Pneumonia/ARI	Quantitative	Community	3	1, 4, 6, 7	1, 4, 5	4
Lindblade (2000)	Uganda	Rural	Malaria	Quantitative	Community	8	1, 2, 8	2, 5	2
Luque (2008)	Ecuador	Rural	Pneumonia/ARI	Mixed methods	Community	4	1	4	1
Makundi (2006)	Tanzania	Unknown	Malaria	Qualitative	Community	5	NA	NA	NA
Malik (2006)	Sudan	Rural	Malaria	Qualitative	Provider	8	NA	NA	NA
Mayombana (2004)	Tanzania	Rural	Malaria	Mixed methods	Community	5	1, 2, 8	1, 4	1
McNee (1995)	Phillipines	Unknown	Pneumonia/ARI	Qualitative	Community	2, 5, 8	NA	NA	NA
Mitiku and Assefa [47]	Ethiopia	Rural + Urban	Malaria	Quantitative	Community	6	1, 2, 5, 8	2	2
Müller et al. [48]	Burkina Faso	Rural + Urban	Malaria	Quantitative	Community	3, 4	1, 2, 7, 8	2	1, 4
Munthali (2003)	Malawi	Rural	Malaria	Qualitative	Community	5	NA	NA	NA
Munthali (2005)	Malawi	Rural	Malaria	Qualitative	Community	5	NA	NA	NA
Muro (2017)	Tanzania	Urban	Pneumonia/ARI	Qualitative	Community + Provider	8	NA	NA	NA
Naheed (2019)	Bangladesh	Rural + Urban	Pneumonia/ARI	Quantitative	Provider	2	1, 4, 7, 8, 10	2	5
Najnin (2011)	Bangladesh	Urban	Fever	Quantitative	Community	4	1, 2, 5, 8	2, 4, 5	2
Nsungwa (2004)	Uganda	Rural	Malaria	Qualitative	Community	5	NA	NA	NA
Okeke (2010a)	Nigeria	Rural	Malaria	Mixed methods	Community	4	1, 2, 5, 8	1, 4	1
Okeke (2010b)	Nigeria	Rural + Urban	Malaria	Qualitative	Community	5	NA	NA	NA
Oluchi et al. [49]	Nigeria	Unknown	Malaria	Quantitative	Community		1, 2, 5, 8	2, 5	2
Onyango (2012)	Kenya	Rural + Urban	Pneumonia/ARI	Quantitative	Provider	2	1, 4, 8, 9	5	5
Rashid (2001)	Bangladesh	Rural	Pneumonia/ARI	Qualitative	Community	4, 5	NA	NA	NA
Salah (2007)	Sudan	Urban	Malaria	Quantitative	Community	5	1	1	1
Sankarapandian (2011)	India	Urban	Pneumonia/ARI	Quantitative	Community	2	1, 5, 8	4	1
Simba (2009)	Tanzania	Rural	Malaria	Quantitative	Provider	2, 4	1, 3, 6, 8, 11	3, 4	2, 3
Simba (2010)	Tanzania	Rural	Malaria	Qualitative	Community	2	NA	NA	NA
Siribié et al. [50]	Burkina Faso, Nigeria, Uganda	Rural	Malaria	Mixed methods	Community + Provider	2	1, 2, 6, 7, 8, 11	3, 4, 5	6
Snavely et al. [51]	Tanzania	Rural + Urban	Fever	Quantitative	Provider	2, 3	1, 2, 7, 8, 10	1, 4, 5	5
Straus 2011	Mozambique	Rural	Pneumonia/ARI	Qualitative	Community + Provider	4	NA	NA	NA
Taffa and Chepngeno [52]	Kenya	Urban	Fever	Quantitative	Community	6	1, 5, 8	2	1
Tarimo (2000)	Tanzania	Rural	Malaria	Quantitative	Provider	4	1, 2, 8, 9	1, 4	1
Thomson (2011)	Sierra Leone	Rural	Malaria	Quantitative	Provider	4	1, 3, 8, 11	3, 5	1
Tinuade et al. [53]	Nigeria	Unknown	Fever	Quantitative	Provider	1, 6	6, 7, 8, 10	5	4
Tsukahara et al. [54]	PNG	Rural	Malaria	Quantitative	Community	6	1, 2, 5, 8	1, 4	2
Ustrup (2014)	Malawi	Rural + Urban	Pneumonia/ARI, Malaria	Quantitative	Community	2	1, 2, 4, 5, 8	2	2
Vaahtera (2000)	Malawi	Rural	Pneumonia/ARI, Malaria	Quantitative	Community	3	2, 4, 5, 7, 8	2	5
Vermeersch (2014)	Guinea-Bissau	Rural	Malaria	Mixed methods	Community	4	1, 6	1, 4	3
Warsame et al. [55]	Tanzania	Rural	Malaria	Qualitative	Provider	2, 4, 5	NA	NA	NA
Yadav (2010)	India	Rural	Malaria	Mixed methods	Provider	4	1, 2, 5, 8, 9	1, 4, 5	1

a SFI definitions: 1 = Clinical management, 2 = Guidelines, 3 = Disease outcome, 4 = Symptoms - biomedical, 5 = Local illness concept, 6 = Perception, 7 = Treatment failure, 8 = Symptoms - caregiver.

b Inclusion criteria: 1 = Age, 2 = (History of) fever, 3 = Malaria diagnosis, 4 = Pneumonia diagnosis, 5 = Recall period, 6 = Severe illness only, 7 = Dead, 8 = Alive, 9 = Attendance (provider enrolment), 10 = Admission (provider enrolment), 11 = Referred (provider enrolment).

c Treatment seeking outcomes: 1 = Treatment seeking proportion, 2 = Dichotomous outcomes, 3 = Referral adherence, 4 = Treatment sources, 5 = Delay.

d Concept of severity: SFI explaining treatment seeking: 1 = Mild vs. severe, no covariates, 2 = Mild vs. severe, with covariates, 3 = Severe only; Treatment seeking explaining SFI: 4 = Severe only, 5 = Mild vs. severe; Absence of causal direction: 6 = Inclusion criteria, no concept of severity.

**TABLE 4:** Categories and definitions of severe febrile illness emerging from included publications (Definitions of severity in treatment seeking studies of febrile illness in children in low and middle income countries: a scoping review, Switzerland, 2021).

Category	Code for SFI definition	Description	Example
Biomedical definitions	Clinical management or diagnosis	The febrile condition requires a specific kind of clinical management unique to severe disease	Tinuade et al. [53]
		The child is diagnosed with severe disease by a clinician (no signs and symptoms specified)	Children with “life-threatening events that required emergency care such as resuscitation, rehydration, blood transfusion, respiratory supports etc.”
	Guidelines	Based on a clinical guideline, the febrile condition is classified as severe	Ellis et al. [18]
			“An illness was labelled as ‘severe febrile illness’ if it met the Integrated Management of Childhood Illness (IMCI) classifications of severe malaria.”
	Disease outcome	Based on the disease outcome, the febrile condition is classified as severe	Kassam et al. [45]
			“(…) the child’s condition progressing from mild to severe, resulting in negative outcomes such as irreversible disability or death.”
	Clinical signs and symptoms	Specific clinical signs and symptoms are considered an indicator of severe disease	Warsame et al. [55]
			“(…) children were admitted to the Regional Hospital with a diagnosis of severe malaria (altered consciousness, coma, convulsions, hypoglycaemia, difficulty in breathing, severe anaemia, prostration).”
Caregiver definitions	Local illness concepts	Local and/or traditional understandings of health conditions perceived to be severe	Beiersmann et al. [34]
			“The local concept of kono is very close to the biomedical definition of cerebral malaria. (…) It is considered a very serious illness which has often fatal outcomes.”
	Perceived severity	Caregivers perceive child’s condition as severe. The perceived severity is not (reported to be) associated to specific symptoms, outcomes or other illness factors	Tsukahara et al. [54]
			“The explanatory variables are (…) severity of the illness as perceived by the caretaker”.
	Perceived treatment failure	Caregivers perceive child’s condition to be severe if treatment fails	Kamat et al. [43]
			“(…) changes in perception of severity (…) based on the failure of home-based treatment”.
	Recognized signs and symptoms	Caregivers perceive specific symptoms or signs of illness to be a sign of severe disease	Chibwana et al. [35]
			“Caregivers had their own way of categorizing fever into mild and severe. (…) febrile children who could not play were considered as having severe fever.”

### Stage 5: Collating, Summarizing, and Reporting the Results

From MaxQDA 2018 Plus, we exported all codes to Microsoft Excel format. After importing into Stata 14 (StataCorp, College Station, TX, United States), we used descriptive statistics to summarize our results.

## Results

### Summary of Included Studies

**FIGURE 1:**
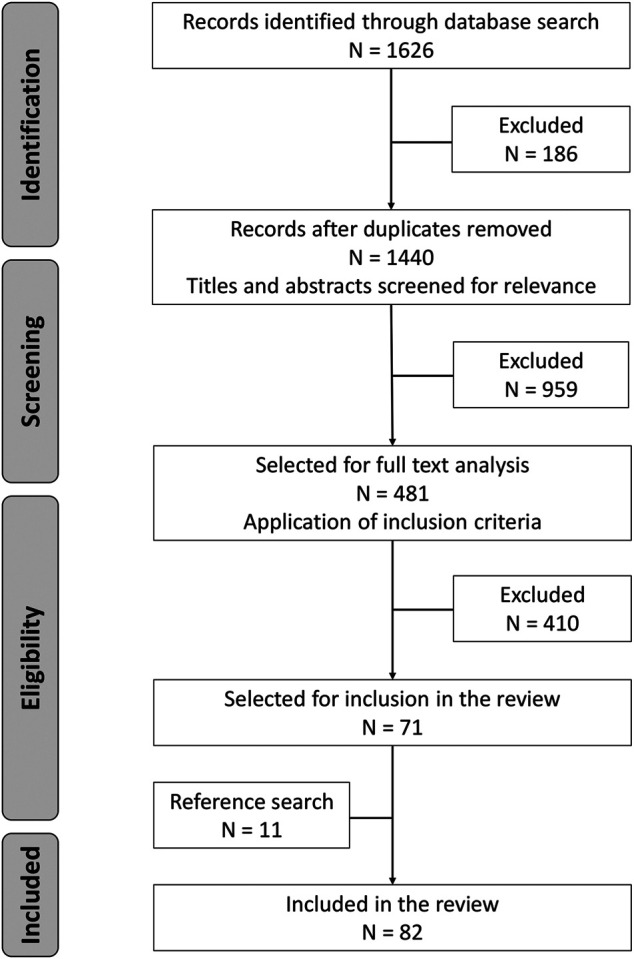
Summary of the literature search and selection process (Definitions of severity in treatment seeking studies of febrile illness in children in low and middle income countries: a scoping review, Switzerland, 2021).

We retrieved 1,626 citations from the three databases. After removing duplicates, the screening process, application of inclusion criteria, and a reference search, we identified 82 studies to be included in the analysis (Figure 1). Main reasons for exclusion in the screening process were 1) the study did not discuss treatment seeking, 2) the study population were mothers instead of children, 3) the study did not distinguish the age group of children under 5 years from other age groups, or 4) the study did not discuss severity. Included manuscripts are listed in Table 3. The reference list for Table 3 is provided in the Supplementary Material S1.

**FIGURE 2:**
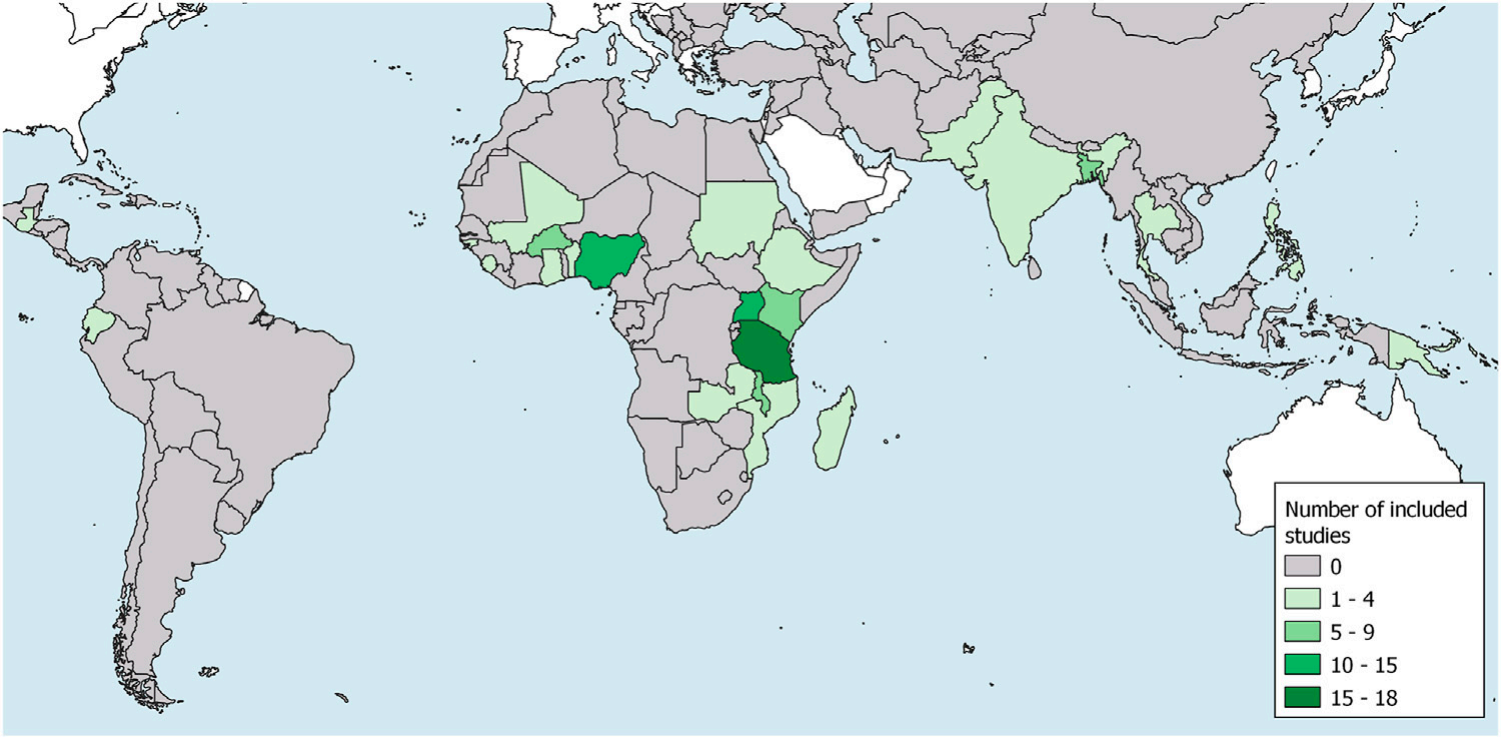
Map showing low and middle income countries with number and location of included studies (Definitions of severity in treatment seeking studies of febrile illness in children in low and middle income countries: a scoping review, Switzerland, 2021).

With the exception of three publication, all selected articles were published from the year 2000 onwards (Table 5). The large majority of studies was conducted in the African Region (82%) (Figure 2). Half were conducted in rural areas (49%). We found that most studies (65%) belonged to the field of malaria research. Other publications mentioned pneumonia or acute respiratory infection (ARI) (32%), in four instances together with malaria. Only one study on meningitis met the inclusion criteria. The remaining six studies (7%) did not refer to any specific disease as the cause for fever in children. The majority of included studies analyzed treatment seeking for SFI using quantitative methods (46%). We identified 27 publications (33%) which discussed treatment seeking for SFI in a purely qualitative way while 17 studies (21%) used a mixed methods approach. Five mixed method studies that explored concepts of SFI in the qualitative component were classified as qualitative SFI study because SFI or treatment seeking did not play a role in the quantitative component of the study. Most studies enrolled their participants from the community (67%). Only six studies (7%) enrolled participants from both community and health care providers. This combination of recruitment strategies was unique to mixed methods and qualitative studies. The median total sample size of quantitative treatment seeking analyses was 304 illness episodes (range: 25–6,856). The respective median sample size of SFI episodes was 112 (range: 12–5,485).

**TABLE 5:** Characteristics of included studies (Definitions of severity in treatment seeking studies of febrile illness in children in low and middle income countries: a scoping review, Switzerland, 2021).

Characteristic	Number of studies (*N* = 82)	Percentage (%)
Publication year		
1990–1994	2	2
1995–1999	1	1
2000–2004	12	15
2005–2009	24	29
2010–2014	21	26
2015–2020	22	27
Study location (WHO Region)		
African Region	67	82
Eastern Mediterranean Region	1	1
South-East Asia Region	10	12
Region of the Americas	3	4
Western Pacific Region	3	4
Context		
Rural	40	49
Urban	15	18
Rural + Urban	22	27
Not specified	6	7
Disease		
Malaria	53	65
Pneumonia/ARI	26	33
Meningitis	1	1
Non-specific	6	7
Method		
Quantitative	38	46
Qualitative	27	33
Mixed methods	17	21
Enrolment		
Community	55	67
Provider	21	26
Community + Provider	6	7

### Definitions of Severe Febrile Illness

We classified definitions of SFI in treatment seeking studies into two main categories (Table 4). The first category defines SFI from a biomedical perspective and comprises four subcategories: clinical management or diagnosis (*n* = 3), guidelines (*n* = 21), disease outcome (*n* = 9), and clinical signs and symptoms classified as severe by the authors of the included study (*n* = 16). Most studies of the category “Guidelines” referred to the Integrated Management of Childhood Illness (IMCI) or Integrated Community Case Management (iCCM) guidelines (16/21 = 76%). Studies which classified specific signs and symptoms as severe were often applying criteria similar to or overlapping with the IMCI/iCCM danger signs. Except for the study by Kassam, et al. [45] which defined negative disease outcome as death or disability, all other studies of this category restricted the definition to death only.

In the second category, we grouped SFI definitions with a focus on the caregiver’s perspective into four subcategories: local illness concepts (*n* = 23), perceived severity (*n* = 13), treatment failure (*n* = 2), and recognized signs and symptoms classified as severe by the caregiver (*n* = 11). Mitiku et al. [47] used a Health Belief Model construct to define perceived severity. Anaba et al. [32] defined perceived severity as caregivers thinking that pneumonia can lead to hospitalization or children may die. The remaining studies did not elaborate on the meaning of perceived severity. In the study by Oluchi, et al. [49], it was not clear which criteria defined severity.

Most studies used only one concept to describe SFI (82%). Using several definitions of SFI occurred in fourteen studies (17%) of which one quantitative, three mixed methods, and four qualitative studies combined a biomedical definition and a definition with a caregiver focus. Overall, 37 included studies (45%) defined SFI from a uniquely biomedical perspective and 36 (44%) uniquely from the perspective of caregivers. The biomedical definitions were more common in quantitative studies (27/38 = 71%) than in qualitative (3/27 = 11%) or mixed methods studies (7/17 = 41%). In quantitative studies that used a caregiver definition (9/38 = 24%), SFI was mostly a febrile illness of high perceived severity (8/9 = 89%). Biomedical definitions were also the predominant definitions in studies enrolling participants from health care providers (12/21 = 57%).

In the process of coding, we found that SFI with convulsions were often treated as a condition distinct from conditions with other symptoms. In ten out of 15 studies that explicitly distinguished convulsions from other conditions, convulsions were the prominent feature of a local illness concept.

### Inclusion and Exclusion Criteria in Quantitative Analyses (*N* = 55)

Out of 55 studies that quantitatively analyzed treatment seeking for SFI in children, 53 studies (96%) restricted the inclusion of children based on age. The most common age group were children aged 0–59 months (69%). The remaining two studies were a cohort of live-born babies and a facility-based study with more than 80% children under 5. Fever or a history of fever was an inclusion criterion in 26 studies (47%). In accordance with the inclusion criteria of this review, 13 studies (24%) included children based on a diagnosis of malaria or pneumonia. Other studies used a vignette which described a febrile condition, enrolled children with any kind of illness but described treatment seeking for fever separately, described a local illness concept that was commonly associated with fever, or fever was not an inclusion criteria but more than 80% of included children were febrile. For community-based studies, it was common to include children that were sick within a specified time period prior to data collection (25/39 = 64%). In most cases, this recall period was 2 weeks (16/25 = 64%). Of those studies with a recall period, two studies from the same first author explicitly excluded children with ongoing illness [37, 38]. The study by Ellis et al. [18] was the only community-based study explicitly including children that were sick at the time of data collection.

Quantitative analyses based on health care provider enrolments (*N* = 14) included children that either attended the health care provider (57%), were admitted (29%), or were referred by the health care provider (14%).

In 16 studies (29%), severity was an inclusion criteria for the analysis of treatment seeking. Most quantitative analyses included study participants irrespective of disease severity but analyzed severe illness separately, or compared treatment seeking for severe illness to treatment seeking for mild illness. Five studies exclusively included dead children (9%). Both dead and alive children were included in ten studies (18%). Inclusion irrespective of health outcome was more common if studies enrolled participants from health care providers (5/14 = 36%) than from communities (4/39 = 10%). The majority of the studies (62%) only included alive children or did not explicitly mention dead children. In nine cases (16%), a vignette was the basis for treatment seeking questions. Three studies also enrolled children with an ongoing illness but used a vignette to increase the sample size, or compare hypothetical with actual treatment seeking.

### Treatment Seeking Outcomes Assessed in Quantitative Analyses (*N* = 55)

Twenty-three studies (42%) distinguished between treatment from outside home and not seeking care from outside home for SFI. Of these, eighteen studies (78%, *N* = 23) further differentiated between different sources of treatment. We found four studies (5%) that reported on referral adherence of which three studies specified to which kind of provider children went after being referred. Other dichotomous outcomes in nineteen studies (34%) were for example “seeking care from a trained/appropriate provider vs all other actions” or “seeking care from a community health worker vs all other actions.” Of those, only one study provided further details on the treatment sources. Overall, 27 studies (49%) specified the treatment sources which consisted of either first providers (*n* = 9), any provider throughout the whole treatment seeking process (*n* = 7), a known sequence of providers (*n* = 7), or providers for which their involvement in the treatment seeking process was not clear (*n* = 4). Mixed methods studies considerably more often specified the treatment sources than quantitative studies [82% (*N* = 17) vs. 34% (*N* = 38)].

Delays were a treatment seeking outcome in 22 studies (40%). Delays were usually defined as a threshold time period (normally 24 h) to reach a health care provider after the onset of symptoms. Four studies used a more complex approach to defining delays by additionally including delays to treatment. In six studies, delay was a continuous variable.

### Concepts of Severity in Quantitative Analyses (*N* = 55)

Quantitative analyses may use several concepts of severity. For example, a study may use two different definitions of SFI and subsequently apply two different concepts of severity, or compare severe illness against mild illness in a first step to subsequently describe treatment seeking for severe illness in more detail without comparison to non-severe disease.

The concept of severity in quantitative analyses did not always become evident from the analysis itself. In some cases, the introduction or discussion of the manuscript had to be consulted to understand whether treatment seeking was considered a consequence of illness severity or vice versa. In two studies, the (sub-) analyses included severely ill children only and severity did not play any further role in explaining treatment seeking or being explained by treatment seeking [30, 50]. In one study, severity both explained treatment seeking and was explained by treatment seeking [48].

#### Severity Explaining Treatment Seeking (*N* = 41)

The majority of the studies (41/55 = 75%) conducted analyses that sought to explain treatment seeking as a consequence of illness severity. Seventeen analyses (44%) compared treatment seeking for SFI against treatment seeking for non-severe illness without additional covariates (often by means of a two-way table). In fifteen analyses (37%), severity was a covariate among other determinants of treatment seeking behavior. Twelve analyses (29%) did not compare against mild conditions but nevertheless understood treatment seeking actions as a consequence of the severe condition of the child.

**FIGURE 3:**
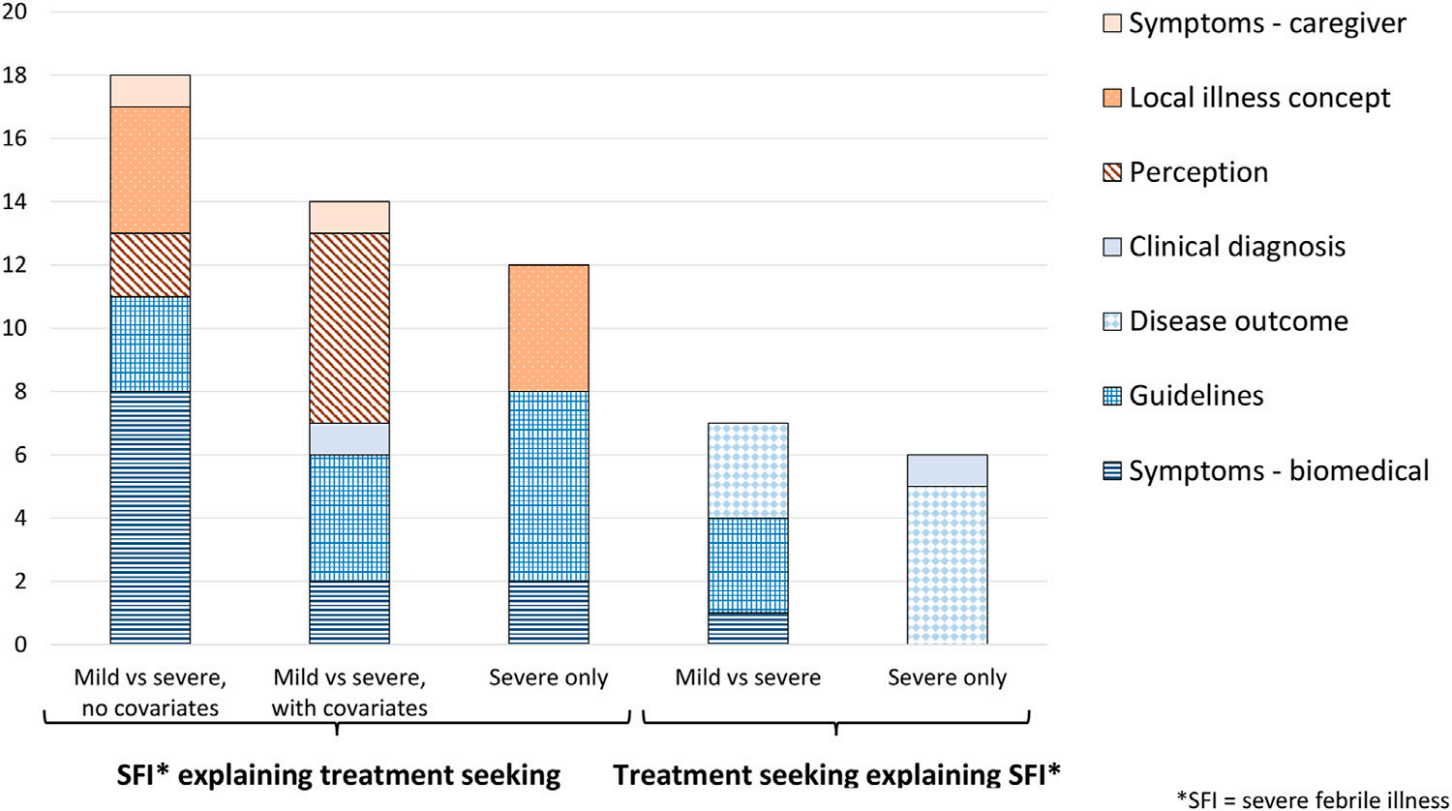
Severe febrile illness definitions in quantitative treatment seeking analyses, by concept of severity and analysis approach (Definitions of severity in treatment seeking studies of febrile illness in children in low and middle income countries: a scoping review, Switzerland, 2021).

Biomedical SFI definitions (*n* = 26) were more common than definitions with a caregiver focus (*n* = 18). Biomedical definitions were either derived from guidelines (*n* = 13), based on symptoms (*n* = 12), or clinical diagnosis (*n* = 1) (Figure 3). Among analyses with a caregiver definition, perceived severity (*n* = 8) and local illness concepts (*n* = 8) were the most frequent SFI definitions before symptoms (*n* = 2). In seven studies, the local illness concept was explored in a qualitative component of a mixed method study.

#### Treatment Seeking Explaining Severity (*N* = 13)

Thirteen studies (24%, *N* = 55) conducted analyses that sought to explain severe illness as a consequence of treatment seeking, e.g., if treatment seeking was delayed or an inappropriate provider was consulted. Seven analyses (54%) compared mild against severe disease to understand the influence of treatment seeking on the development of severe illness. In six analyses (46%) only severe cases were included but the study narrative was clear about the causal direction of the relationship between treatment seeking and severe illness.

Only biomedical definitions of SFI were used of which disease outcome, i.e., death, was the most common one (*n* = 8) (Figure 3).

## Discussion

In this scoping review, we explored the definitions of SFI in treatment seeking studies and investigated concepts of severity in quantitative analyses of treatment seeking behavior. The definitions of SFI differed between quantitative and qualitative studies, and depended on the direction of the causal pathway between illness severity and treatment seeking in quantitative analyses. Our findings suggest that concerning treatment seeking outcomes and the definition of SFI two perspectives exist.

The first perspective is normative, i.e., what people should do, and emerges from the idea of a health system that allocates resources for rational and efficient use [56, 57, 58]. Accordingly, we found that treatment seeking is often dichotomized into right and wrong actions, e.g., seeking care from an appropriate or trained provider versus all other actions. Consequently, children who sought treatment from an inappropriate or untrained provider belong to the same category as children who did not seek care at all. This normative simplification of the treatment seeking process was also present in studies which only report the first provider after illness onset or referral, studies which measure delay to provider attendance (instead of treatment), studies which dichotomize delay into early and late, and one study on referral adherence which did not specify the source of subsequent treatment. Besides normative assumptions, the relative rare occurrence of severe illness, reflected by the smaller size of SFI (sub-) samples, could be a reason why many studies aggregate treatment seeking outcomes to detect statistical significant differences. However, depending on the research question, this normative approach increases the risk of oversimplifying human behavior.

Similarly, we found biomedical definitions of SFI to be of normative nature. The IMCI/iCCM algorithm offers clear instructions on treatment and referral to higher level facilities for cases of SFI [59]. While death is not necessarily normative, its occurrence in children is a clear indication that medical care should have been sought and provided. A systematic review by Price et al. [10] used the Pathways to Survival framework to explore modifiable factors of the care seeking process contributing to death. The Pathways to Survival framework uses similar steps in the treatment seeking process that above have been described as normative [60]. Both examples show that connecting illness definitions with a normative understanding of treatment seeking induces a normative understanding of the illness itself.

In quantitative studies, biomedical definitions are predominant. This approach may be the preferred choice when treatment seeking explains severe illness. With increasing severity of the health outcome, understanding the circumstances that lead to severe disease becomes more important and relevant [10]. Congruently, most of the analyses in studies which sought to explain the development of SFI with treatment seeking defined SFI as death. However, when a severe presentation of illness is used to explain treatment seeking a caregiver definition might be more appropriate. Local understanding of disease does not always overlap with biomedical definitions, despite previous research showing that caregivers are able to recognize symptoms indicating severe disease [7, 61]. The tendency to ignore the caregiver perspective in analyses conceptualizing treatment seeking as a reaction to SFI may be a consequence of the lack of involvement of social scientists in treatment seeking studies [6]. Treatment seeking studies are conducted with different intentions: informing the implementation of an intervention [31, 33, 44], assessing the effectiveness of an intervention [40, 41], and investigating barriers to health care [18, 42, 47] among others. These intentions should be reflected in the definitions, concepts and methodological approaches of a study.

The second perspective is descriptive, i.e., what people in fact do, and takes into consideration caregivers’ need to seek treatment for their children in a context of limited resources [6, 57]. This descriptive approach portrays treatment seeking as a process involving multiple providers with changing sequence and several stages of delay. Given its descriptive and inductive nature, complete treatment seeking narratives are more common in qualitative research [6, 62]. However, we found several examples of quantitative analyses from mostly mixed methods studies that were able to capture the complexities of complete treatment seeking pathways [18, 33, 36, 51].

Correspondingly, the non-normative approach to define SFI are caregiver definitions. While these definitions were most common in qualitative studies, a considerable number of quantitative analyses conceptualizing treatment seeking as a consequence of SFI used a caregiver definition. One of the predominant caregiver definitions was perceived severity. Except for two studies, none of the included studies elaborated on the meaning of perceived severity. Several health behavior theories emphasize the multidimensionality of perceived severity which may take into consideration emotions, health consequences including death, disease knowledge, and effects on social and work life including economic consequences [5, 20, 21]. The failure of quantitative studies to explore the meaning of perceived severity may explain why some studies find an association between perceived severity and treatment seeking [44, 52, 54] while others do not [39, 46, 53]. Additionally, recall periods are a common feature of quantitative treatment studies. Depending on the disease outcome and experiences with health care providers, caregivers may reevaluate the illness of their child throughout the treatment seeking process and thereafter [45, 63, 64]. Therefore, the perceived severity reported several weeks after the illness episode may not reflect the caregiver’s perception during the period of treatment seeking. To maximize the value of a caregiver definition of SFI, the underlying assumptions need to be well understood and if necessary explored within the context of the same study.

Mixed method studies were outstanding in several ways. Combining qualitative and quantitative methods allows to explore the local meaning of SFI and to understand the importance of different health providers as treatment sources for this illness category. Some studies failed to make use of this major advantage of a mixed methods approach, using different SFI definitions in the qualitative and quantitative components, or exploring a local illness concept in the qualitative component and ignoring it in the quantitative part. Additionally, we found that mixed method studies more often specify the treatment sources than quantitative studies. Even though this approach does not depend on the combination of qualitative and quantitative methods, it might be an indication of the involvement of social scientists who employ a less normative understanding of the complexities of human behavior [6].

We found several indications of a lack of methodological rigor which has been a concern in previous reviews of treatment seeking for malaria [6, 65]. First, the stage of the treatment seeking pathway at which a specific health care provider was consulted was not always clear. To understand the complex and iterative process of treatment seeking, studies need to be transparent about the stages of the treatment seeking process being part of the research and which types of provider were consulted.

Secondly, retrospective community-based studies often fail to explicitly state the inclusion or exclusion of dead children or children with ongoing illness. Besides ethical concerns, the inclusion of children with ongoing illness whose treatment seeking pathway may not have been finished, and the exclusion of dead children whose treatment seeking pathways may be different from surviving children increases the risk of selection bias. By systematically excluding dead children in cross-sectional surveys, problematic treatment seeking pathways at community level remain hidden and generated evidence may portray an unrealistically positive picture of local treatment seeking practices. In general, treatment seeking studies including dead and alive children are scarce resulting in a lack of evidence to understand the differences in treatment seeking pathways between surviving children and children that die, and hence, in a more general sense, the association of treatment seeking and health outcomes [10].

An additional concern is the relative abundance of treatment seeking studies on mild illness compared to severe illness [6, 8]. Demographic and Health Surveys, Malaria Indicator Surveys, and Multiple Cluster Indicator Surveys collect quantitative data on treatment seeking for fever in children in a majority of malaria-endemic countries but do not consider severity as an explanatory variable [66]. Adding a severity indicator to large-scale surveys would increase the availability of data on SFI and related treatment seeking behavior. A challenge remains the definition of a meaningful severity indicator applicable to a wide range of contexts to allow for cross-country comparisons.

### Limitations

The methodology of this review has several limitations. Due to the diversity in definitions of severity in treatment seeking studies, we conducted a scoping review instead of a systematic review. Unlike a systematic review, scoping reviews do not allow for the synthetization of evidence and aggregation of findings from the body of literature [22]. Instead, we summarize the range of research and identify research gaps. Concerning treatment seeking outcomes, the scope of our findings is limited by the exclusion of studies on treatment seeking for non-severe febrile illness. The frequency and range of treatment seeking outcomes might be different in studies on mild illness. Malaria and pneumonia are two of the most common febrile illnesses in children. Including them in the search strategy may have led to an overrepresentation of these illnesses and an underrepresentation of less common conditions. Other SFIs might concern adults as much as children and treatment seeking studies may not differentiate between age groups. Restricting the inclusion criteria to children under 5 may have introduced additional bias towards malaria and pneumonia. Fever has commonly been used as a proxy for malaria in treatment seeking studies in areas of high malaria transmission. With a reduction of transmission, non-malarial fevers will become more important [67]. This epidemiological transition might impact the association between treatment seeking and health outcomes of SFI, but also emphasizes the importance of a syndromic approach to understanding treatment seeking for SFI and fever in general.

### Conclusion

The diversity of SFI definitions and treatment seeking outcomes in treatment seeking studies of febrile illness in children under five affects the comparability of the evidence in this field of research. However, we do not recommend a single definition or approach. For researchers who plan to conduct a treatment seeking study, the focus should lie on defining the intention of their study and considering the different perspectives on illness concepts and treatment seeking relevant to their research. A non-normative understanding of illness and human behavior is particularly important when little is known about treatment seeking practices in the local communities and when interventions aiming at improving health practices and service provision need to be adapted to the local context. Quantitative research should apply knowledge gained through qualitative research and only be guided by normative assumptions when a health system perspective is more relevant than a community perspective.

The lack of methodological rigor remains a concern in treatment seeking studies. Limited information on inclusion and exclusion criteria, and ambiguities in reported treatment seeking outcomes make it difficult to draw conclusions from the presented evidence and to make comparisons between studies. Due to limited information on the inclusion of dead children and study designs excluding dead children there is dearth of evidence on the differences in treatment seeking pathways between children who survive and recover and children who die. This limits the validity of inferences made on the association of treatment seeking pathways and health outcomes in children with SFI. High-quality community studies are needed to fill this research gap.
