# Erratum: Definitions of Severity in Treatment Seeking Studies of Febrile Illness in Children in Low and Middle Income Countries: A Scoping Review

**DOI:** 10.3389/ijph.2021.1604518

**Published:** 2021-12-16

**Authors:** Swiss School of Public Health Production Office

**Affiliations:** Frontiers Media SA, Lausanne, Switzerland

**Keywords:** fever, severity, child, developing countries, treatment seeking, health behavior

Due to a production error, all citations in the text between references 17 and 61 were incorrect. In the **Introduction**, the second citation of reference 17 was incorrectly written as reference 18, thereby shifting all subsequent references by one place.

The text in **Paragraph 3** of the **Introduction** has been updated from “The concept of SFI has been referred to by several studies. Even though Roddy et al. [17] describe SFI as “a commonly documented and globally recognized cause of morbidity and mortality,” definitions of SFI in the scientific literature vary. While some authors have focused on clinical diagnosis [13, 18] or causal agents [16], others defined SFI as fever with danger signs according to the Integrated Management of Childhood Illness (IMCI) guidelines [19]. Health behavior theories have also pointed out the importance of perceived severity for health-related behavior such as treatment seeking [20-22].” to “The concept of SFI has been referred to by several studies. Even though Roddy et al. [17] describe SFI as “a commonly documented and globally recognized cause of morbidity and mortality,” definitions of SFI in the scientific literature vary. While some authors have focused on clinical diagnosis [13, 17] or causal agents [16], others defined SFI as fever with danger signs according to the Integrated Management of Childhood Illness (IMCI) guidelines [18]. Health behavior theories have also pointed out the importance of perceived severity for health-related behavior such as treatment seeking [19-21].”

All subsequent citations have updated to account for this shift.

The publisher apologizes for this mistake. The original version of this article has been updated.

