# Supplementary material for: Definitions of Severity in Treatment Seeking Studies of Febrile Illness in Children in Low and Middle Income Countries: A Scoping Review
Source: Int J Public Health. 2021 Aug 30;66:634000. doi: 10.3389/ijph.2021.634000 (PMC8435535; doi:10.3389/ijph.2021.634000)
Supplement: Supplementary file 1 [file DataSheet1.docx]

Supplementary Material

# Supplementary Tables

**Supplementary Table 1:** Search strategy and results for searched databases (Definitions of severity in treatment seeking studies of febrile illness in children in low and middle income countries: a scoping review, Switzerland, 2021).

| **Database** | **Search terms** | **No. of papers retrieved** |
| --- | --- | --- |
| PubMed | (health seeking[tiab] OR health-seeking[tiab] OR care seeking[tiab] OR care-seeking[tiab] OR healthcare seeking[tiab] OR healthcare-seeking[tiab] OR treatment seeking[tiab] OR treatment-seeking[tiab] OR health behaviour[tiab] OR health behavior[tiab] OR health care behaviour[tiab] OR health care behavior[tiab] OR healthcare behaviour[tiab] OR healthcare behavior[tiab] OR illness behaviour[tiab] OR illness behavior[tiab] OR health service utilisation[tiab] OR health service utilization[tiab] OR health care use[tiab] OR healthcare use[tiab]) OR (Health Knowledge, Attitudes, Practice[Mesh] OR Patient Acceptance of Health Care[Mesh])  AND (fever*[tiab] OR febrile[tiab] OR fever[mesh] OR hyperthermia[tiab] OR pyrexi*[tiab] OR malaria[tiab] or malaria[mesh] or pneumonia[tiab] or pneumonia[mesh])  AND (Infan*[tiab] OR newborn*[tiab] OR new-born*[tiab] OR perinat*[tiab] OR neonat*[tiab] OR baby*[tiab] OR babies[tiab] OR toddler*[tiab] OR minors*[tiab] OR boy*[tiab] OR girl*[tiab] OR kid[tiab] OR kids[tiab] OR child*[tiab] OR pediatrics[mh] OR pediatric*[tiab] OR paediatric*[tiab] OR peadiatric*[tiab])  AND ("developing country"[tiab] OR "developing countries"[tiab] OR "developing nation"[tiab] OR "developing nations"[tiab] OR "developing population"[tiab] OR "developing populations"[tiab] OR "developing world"[tiab] OR "less developed country"[tiab] OR "less developed countries"[tiab] OR "less developed nation"[tiab] OR "less developed nations"[tiab] OR "less developed population"[tiab] OR "less developed populations"[tiab] OR "less developed world"[tiab] OR "lesser developed country"[tiab] OR "lesser developed countries"[tiab] OR "lesser developed nation"[tiab] OR "lesser developed nations"[tiab] OR "lesser developed population"[tiab] OR "lesser developed populations"[tiab] OR "lesser developed world"[tiab] OR "under developed country"[tiab] OR "under developed countries"[tiab] OR "under developed nation"[tiab] OR "under developed nations"[tiab] OR "under developed population"[tiab] OR "under developed populations"[tiab] OR "under developed world"[tiab] OR "underdeveloped country"[tiab] OR "underdeveloped countries"[tiab] OR "underdeveloped nation"[tiab] OR "underdeveloped nations"[tiab] OR "underdeveloped population"[tiab] OR "underdeveloped populations"[tiab] OR "underdeveloped world"[tiab] OR "middle income country"[tiab] OR "middle income countries"[tiab] OR "middle income nation"[tiab] OR "middle income nations"[tiab] OR "middle income population"[tiab] OR "middle income populations"[tiab] OR "low income country"[tiab] OR "low income countries"[tiab] OR "low income nation"[tiab] OR "low income nations"[tiab] OR "low income population"[tiab] OR "low income populations"[tiab] OR "lower income country"[tiab] OR "lower income countries"[tiab] OR "lower income nation"[tiab] OR "lower income nations"[tiab] OR "lower income population"[tiab] OR "lower income populations"[tiab] OR "underserved country"[tiab] OR "underserved countries"[tiab] OR "underserved nation"[tiab] OR "underserved nations"[tiab] OR "underserved population"[tiab] OR "underserved populations"[tiab] OR "underserved world"[tiab] OR "under served country"[tiab] OR "under served countries"[tiab] OR "under served nation"[tiab] OR "under served nations"[tiab] OR "under served population"[tiab] OR "under served populations"[tiab] OR "under served world"[tiab] OR "deprived country"[tiab] OR "deprived countries"[tiab] OR "deprived nation"[tiab] OR "deprived nations"[tiab] OR "deprived population"[tiab] OR "deprived populations"[tiab] OR "deprived world"[tiab] OR "poor country"[tiab] OR "poor countries"[tiab] OR "poor nation"[tiab] OR "poor nations"[tiab] OR "poor population"[tiab] OR "poor populations"[tiab] OR "poor world"[tiab] OR "poorer country"[tiab] OR "poorer countries"[tiab] OR "poorer nation"[tiab] OR "poorer nations"[tiab] OR "poorer population"[tiab] OR "poorer populations"[tiab] OR "poorer world"[tiab] OR "developing economy"[tiab] OR "developing economies"[tiab] OR "less developed economy"[tiab] OR "less developed economies"[tiab] OR "lesser developed economy"[tiab] OR "lesser developed economies"[tiab] OR "under developed economy"[tiab] OR "under developed economies"[tiab] OR "underdeveloped economy"[tiab] OR "underdeveloped economies"[tiab] OR "middle income economy"[tiab] OR "middle income economies"[tiab] OR "low income economy"[tiab] OR "low income economies"[tiab] OR "lower income economy"[tiab] OR "lower income economies"[tiab] OR "low gdp"[tiab] OR "low gnp"[tiab] OR "low gross domestic"[tiab] OR "low gross national"[tiab] OR "lower gdp"[tiab] OR "lower gnp"[tiab] OR "lower gross domestic"[tiab] OR "lower gross national"[tiab] OR lmic[tiab] OR lmics[tiab] OR "third world"[tiab] OR "lami country"[tiab] OR "lami countries"[tiab] OR "transitional country"[tiab] OR "transitional countries"[tiab]) OR("developing country"[ot] OR "developing countries"[ot] OR "developing nation"[ot] OR "developing nations"[ot] OR "developing population"[ot] OR "developing populations"[ot] OR "developing world"[ot] OR "less developed country"[ot] OR "less developed countries"[ot] OR "less developed nation"[ot] OR "less developed nations"[ot] OR "less developed population"[ot] OR "less developed populations"[ot] OR "less developed world"[ot] OR "lesser developed country"[ot] OR "lesser developed countries"[ot] OR "lesser developed nation"[ot] OR "lesser developed nations"[ot] OR "lesser developed population"[ot] OR "lesser developed populations"[ot] OR "lesser developed world"[ot] OR "under developed country"[ot] OR "under developed countries"[ot] OR "under developed nation"[ot] OR "under developed nations"[ot] OR "under developed population"[ot] OR "under developed populations"[ot] OR "under developed world"[ot] OR "underdeveloped country"[ot] OR "underdeveloped countries"[ot] OR "underdeveloped nation"[ot] OR "underdeveloped nations"[ot] OR "underdeveloped population"[ot] OR "underdeveloped populations"[ot] OR "underdeveloped world"[ot] OR "middle income country"[ot] OR "middle income countries"[ot] OR "middle income nation"[ot] OR "middle income nations"[ot] OR "middle income population"[ot] OR "middle income populations"[ot] OR "low income country"[ot] OR "low income countries"[ot] OR "low income nation"[ot] OR "low income nations"[ot] OR "low income population"[ot] OR "low income populations"[ot] OR "lower income country"[ot] OR "lower income countries"[ot] OR "lower income nation"[ot] OR "lower income nations"[ot] OR "lower income population"[ot] OR "lower income populations"[ot] OR "underserved country"[ot] OR "underserved countries"[ot] OR "underserved nation"[ot] OR "underserved nations"[ot] OR "underserved population"[ot] OR "underserved populations"[ot] OR "underserved world"[ot] OR "under served country"[ot] OR "under served countries"[ot] OR "under served nation"[ot] OR "under served nations"[ot] OR "under served population"[ot] OR "under served populations"[ot] OR "under served world"[ot] OR "deprived country"[ot] OR "deprived countries"[ot] OR "deprived nation"[ot] OR "deprived nations"[ot] OR "deprived population"[ot] OR "deprived populations"[ot] OR "deprived world"[ot] OR "poor country"[ot] OR "poor countries"[ot] OR "poor nation"[ot] OR "poor nations"[ot] OR "poor population"[ot] OR "poor populations"[ot] OR "poor world"[ot] OR "poorer country"[ot] OR "poorer countries"[ot] OR "poorer nation"[ot] OR "poorer nations"[ot] OR "poorer population"[ot] OR "poorer populations"[ot] OR "poorer world"[ot] OR "developing economy"[ot] OR "developing economies"[ot] OR "less developed economy"[ot] OR "less developed economies"[ot] OR "lesser developed economy"[ot] OR "lesser developed economies"[ot] OR "under developed economy"[ot] OR "under developed economies"[ot] OR "underdeveloped economy"[ot] OR "underdeveloped economies"[ot] OR "middle income economy"[ot] OR "middle income economies"[ot] OR "low income economy"[ot] OR "low income economies"[ot] OR "lower income economy"[ot] OR "lower income economies"[ot] OR "low gdp"[ot] OR "low gnp"[ot] OR "low gross domestic"[ot] OR "low gross national"[ot] OR "lower gdp"[ot] OR "lower gnp"[ot] OR "lower gross domestic"[ot] OR "lower gross national"[ot] OR lmic[ot] OR lmics[ot] OR "third world"[ot] OR "lami country"[ot] OR "lami countries"[ot] OR "transitional country"[ot] OR "transitional countries"[ot]) OR(Africa[tiab] OR Asia[tiab] OR Caribbean[tiab] OR West Indies[tiab] OR South America[tiab] OR Latin America[tiab] OR Central America[tiab] OR Afghanistan[tiab] OR Albania[tiab] OR Algeria[tiab] OR Angola[tiab] OR Antigua[tiab] OR Barbuda[tiab] OR Argentina[tiab] OR Armenia[tiab] OR Armenian[tiab] OR Aruba[tiab] OR Azerbaijan[tiab] OR Bahrain[tiab] OR Bangladesh[tiab] OR Barbados[tiab] OR Benin[tiab] OR Byelarus[tiab] OR Byelorussian[tiab] OR Belarus[tiab] OR Belorussian[tiab] OR Belorussia[tiab] OR Belize[tiab] OR Bhutan[tiab] OR Bolivia[tiab] OR Bosnia[tiab] OR Herzegovina[tiab] OR Hercegovina[tiab] OR Botswana[tiab] OR Brasil[tiab] OR Brazil[tiab] OR Bulgaria[tiab] OR Burkina Faso[tiab] OR Burkina Fasso[tiab] OR Upper Volta[tiab] OR Burundi[tiab] OR Urundi[tiab] OR Cambodia[tiab] OR Khmer Republic[tiab] OR Kampuchea[tiab] OR Cameroon[tiab] OR Cameroons[tiab] OR Cameron[tiab] OR Camerons[tiab] OR Cape Verde[tiab] OR Central African Republic[tiab] OR Chad[tiab] OR Chile[tiab] OR China[tiab] OR Colombia[tiab] OR Comoros[tiab] OR Comoro Islands[tiab] OR Comores[tiab] OR Mayotte[tiab] OR Congo[tiab] OR Zaire[tiab] OR Costa Rica[tiab] OR Cote d'Ivoire[tiab] OR Ivory Coast[tiab] OR Croatia[tiab] OR Cuba[tiab] OR Cyprus[tiab] OR Czechoslovakia[tiab] OR Czech Republic[tiab] OR Slovakia[tiab] OR Slovak Republic[tiab] OR Djibouti[tiab] OR French Somaliland[tiab] OR Dominica[tiab] OR Dominican Republic[tiab] OR East Timor[tiab] OR East Timur[tiab] OR Timor Leste[tiab] OR Ecuador[tiab] OR Egypt[tiab] OR United Arab Republic[tiab] OR El Salvador[tiab] OR Eritrea[tiab] OR Estonia[tiab] OR Ethiopia[tiab] OR Fiji[tiab] OR Gabon[tiab] OR Gabonese Republic[tiab] OR Gambia[tiab] OR Gaza[tiab] OR Georgia Republic[tiab] OR Georgian Republic[tiab] OR Ghana[tiab] OR Gold Coast[tiab] OR Greece[tiab] OR Grenada[tiab] OR Guatemala[tiab] OR Guinea[tiab] OR Guam[tiab] OR Guiana[tiab] OR Guyana[tiab] OR Haiti[tiab] OR Honduras[tiab] OR Hungary[tiab] OR India[tiab] OR Maldives[tiab] OR Indonesia[tiab] OR Iran[tiab] OR Iraq[tiab] OR Isle of Man[tiab] OR Jamaica[tiab] OR Jordan[tiab] OR Kazakhstan[tiab] OR Kazakh[tiab] OR Kenya[tiab] OR Kiribati[tiab] OR Korea[tiab] OR Kosovo[tiab] OR Kyrgyzstan[tiab] OR Kirghizia[tiab] OR Kyrgyz Republic[tiab] OR Kirghiz[tiab] OR Kirgizstan[tiab] OR "Lao PDR"[tiab] OR Laos[tiab] OR Latvia[tiab] OR Lebanon[tiab] OR Lesotho[tiab] OR Basutoland[tiab] OR Liberia[tiab] OR Libya[tiab] OR Lithuania[tiab]) OR(Macedonia[tiab] OR Madagascar[tiab] OR Malagasy Republic[tiab] OR Malaysia[tiab] OR Malaya[tiab] OR Malay[tiab] OR Sabah[tiab] OR Sarawak[tiab] OR Malawi[tiab] OR Nyasaland[tiab] OR Mali[tiab] OR Malta[tiab] OR Marshall Islands[tiab] OR Mauritania[tiab] OR Mauritius[tiab] OR Agalega Islands[tiab] OR Mexico[tiab] OR Micronesia[tiab] OR Middle East[tiab] OR Moldova[tiab] OR Moldovia[tiab] OR Moldovian[tiab] OR Mongolia[tiab] OR Montenegro[tiab] OR Morocco[tiab] OR Ifni[tiab] OR Mozambique[tiab] OR Myanmar[tiab] OR Myanma[tiab] OR Burma[tiab] OR Namibia[tiab] OR Nepal[tiab] OR Netherlands Antilles[tiab] OR New Caledonia[tiab] OR Nicaragua[tiab] OR Niger[tiab] OR Nigeria[tiab] OR Northern Mariana Islands[tiab] OR Oman[tiab] OR Muscat[tiab] OR Pakistan[tiab] OR Palau[tiab] OR Palestine[tiab] OR Panama[tiab] OR Paraguay[tiab] OR Peru[tiab] OR Philippines[tiab] OR Philipines[tiab] OR Phillipines[tiab] OR Phillippines[tiab] OR Poland[tiab] OR Portugal[tiab] OR Puerto Rico[tiab] OR Romania[tiab] OR Rumania[tiab] OR Roumania[tiab] OR Russia[tiab] OR Russian[tiab] OR Rwanda[tiab] OR Ruanda[tiab] OR Saint Kitts[tiab] OR St Kitts[tiab] OR Nevis[tiab] OR Saint Lucia[tiab] OR St Lucia[tiab] OR Saint Vincent[tiab] OR St Vincent[tiab] OR Grenadines[tiab] OR Samoa[tiab] OR Samoan Islands[tiab] OR Navigator Island[tiab] OR Navigator Islands[tiab] OR Sao Tome[tiab] OR Saudi Arabia[tiab] OR Senegal[tiab] OR Serbia[tiab] OR Montenegro[tiab] OR Seychelles[tiab] OR Sierra Leone[tiab] OR Slovenia[tiab] OR Sri Lanka[tiab] OR Ceylon[tiab] OR Solomon Islands[tiab] OR Somalia[tiab] OR Sudan[tiab] OR Suriname[tiab] OR Surinam[tiab] OR Swaziland[tiab] OR Syria[tiab] OR Tajikistan[tiab] OR Tadzhikistan[tiab] OR Tadjikistan[tiab] OR Tadzhik[tiab] OR Tanzania[tiab] OR Thailand[tiab] OR Togo[tiab] OR Togolese Republic[tiab] OR Tonga[tiab] OR Trinidad[tiab] OR Tobago[tiab] OR Tunisia[tiab] OR Turkey[tiab] OR Turkmenistan[tiab] OR Turkmen[tiab] OR Uganda[tiab] OR Ukraine[tiab] OR Uruguay[tiab] OR USSR[tiab] OR Soviet Union[tiab] OR Union of Soviet Socialist Republics[tiab] OR Uzbekistan[tiab] OR Uzbek OR Vanuatu[tiab] OR New Hebrides[tiab] OR Venezuela[tiab] OR Vietnam[tiab] OR Viet Nam[tiab] OR West Bank[tiab] OR Yemen[tiab] OR Yugoslavia[tiab] OR Zambia[tiab] OR Zimbabwe[tiab] OR Rhodesia[tiab]) OR(Africa[ot] OR Asia[ot] OR Caribbean[ot] OR West Indies[ot] OR South America[ot] OR Latin America[ot] OR Central America[ot] OR Afghanistan[ot] OR Albania[ot] OR Algeria[ot] OR Angola[ot] OR Antigua[ot] OR Barbuda[ot] OR Argentina[ot] OR Armenia[ot] OR Armenian[ot] OR Aruba[ot] OR Azerbaijan[ot] OR Bahrain[ot] OR Bangladesh[ot] OR Barbados[ot] OR Benin[ot] OR Byelarus[ot] OR Byelorussian[ot] OR Belarus[ot] OR Belorussian[ot] OR Belorussia[ot] OR Belize[ot] OR Bhutan[ot] OR Bolivia[ot] OR Bosnia[ot] OR Herzegovina[ot] OR Hercegovina[ot] OR Botswana[ot] OR Brasil[ot] OR Brazil[ot] OR Bulgaria[ot] OR Burkina Faso[ot] OR Burkina Fasso[ot] OR Upper Volta[ot] OR Burundi[ot] OR Urundi[ot] OR Cambodia[ot] OR Khmer Republic[ot] OR Kampuchea[ot] OR Cameroon[ot] OR Cameroons[ot] OR Cameron[ot] OR Camerons[ot] OR Cape Verde[ot] OR Central African Republic[ot] OR Chad[ot] OR Chile[ot] OR China[ot] OR Colombia[ot] OR Comoros[ot] OR Comoro Islands[ot] OR Comores[ot] OR Mayotte[ot] OR Congo[ot] OR Zaire[ot] OR Costa Rica[ot] OR Cote d'Ivoire[ot] OR Ivory Coast[ot] OR Croatia[ot] OR Cuba[ot] OR Cyprus[ot] OR Czechoslovakia[ot] OR Czech Republic[ot] OR Slovakia[ot] OR Slovak Republic[ot] OR Djibouti[ot] OR French Somaliland[ot] OR Dominica[ot] OR Dominican Republic[ot] OR East Timor[ot] OR East Timur[ot] OR Timor Leste[ot] OR Ecuador[ot] OR Egypt[ot] OR United Arab Republic[ot] OR El Salvador[ot] OR Eritrea[ot] OR Estonia[ot] OR Ethiopia[ot] OR Fiji[ot] OR Gabon[ot] OR Gabonese Republic[ot] OR Gambia[ot] OR Gaza[ot] OR "Georgia Republic"[ot] OR "Georgian Republic"[ot] OR Ghana[ot] OR Gold Coast[ot] OR Greece[ot] OR Grenada[ot] OR Guatemala[ot] OR Guinea[ot] OR Guam[ot] OR Guiana[ot] OR Guyana[ot] OR Haiti[ot] OR Honduras[ot] OR Hungary[ot] OR India[ot] OR Maldives[ot] OR Indonesia[ot] OR Iran[ot] OR Iraq[ot] OR Isle of Man[ot] OR Jamaica[ot] OR Jordan[ot] OR Kazakhstan[ot] OR Kazakh[ot] OR Kenya[ot] OR Kiribati[ot] OR Korea[ot] OR Kosovo[ot] OR Kyrgyzstan[ot] OR Kirghizia[ot] OR Kyrgyz Republic[ot] OR Kirghiz[ot] OR Kirgizstan[ot] OR "Lao PDR"[ot] OR Laos[ot] OR Latvia[ot] OR Lebanon[ot] OR Lesotho[ot] OR Basutoland[ot] OR Liberia[ot] OR Libya[ot] OR Lithuania[ot]) OR(Macedonia[ot] OR Madagascar[ot] OR Malagasy Republic[ot] OR Malaysia[ot] OR Malaya[ot] OR Malay[ot] OR Sabah[ot] OR Sarawak[ot] OR Malawi[ot] OR Nyasaland[ot] OR Mali[ot] OR Malta[ot] OR Marshall Islands[ot] OR Mauritania[ot] OR Mauritius[ot] OR Agalega Islands[ot] OR Mexico[ot] OR Micronesia[ot] OR Middle East[ot] OR Moldova[ot] OR Moldovia[ot] OR Moldovian[ot] OR Mongolia[ot] OR Montenegro[ot] OR Morocco[ot] OR Ifni[ot] OR Mozambique[ot] OR Myanmar[ot] OR Myanma[ot] OR Burma[ot] OR Namibia[ot] OR Nepal[ot] OR Netherlands Antilles[ot] OR New Caledonia[ot] OR Nicaragua[ot] OR Niger[ot] OR Nigeria[ot] OR Northern Mariana Islands[ot] OR Oman[ot] OR Muscat[ot] OR Pakistan[ot] OR Palau[ot] OR Palestine[ot] OR Panama[ot] OR Paraguay[ot] OR Peru[ot] OR Philippines[ot] OR Philipines[ot] OR Phillipines[ot] OR Phillippines[ot] OR Poland[ot] OR Portugal[ot] OR Puerto Rico[ot] OR Romania[ot] OR Rumania[ot] OR Roumania[ot] OR Russia[ot] OR Russian[ot] OR Rwanda[ot] OR Ruanda[ot] OR Saint Kitts[ot] OR St Kitts[ot] OR Nevis[ot] OR Saint Lucia[ot] OR St Lucia[ot] OR Saint Vincent[ot] OR St Vincent[ot] OR Grenadines[ot] OR Samoa[ot] OR Samoan Islands[ot] OR Navigator Island[ot] OR Navigator Islands[ot] OR Sao Tome[ot] OR Saudi Arabia[ot] OR Senegal[ot] OR Serbia[ot] OR Montenegro[ot] OR Seychelles[ot] OR Sierra Leone[ot] OR Slovenia[ot] OR Sri Lanka[ot] OR Ceylon[ot] OR Solomon Islands[ot] OR Somalia[ot] OR Sudan[ot] OR Suriname[ot] OR Surinam[ot] OR Swaziland[ot] OR Syria[ot] OR Tajikistan[ot] OR Tadzhikistan[ot] OR Tadjikistan[ot] OR Tadzhik[ot] OR Tanzania[ot] OR Thailand[ot] OR Togo[ot] OR Togolese Republic[ot] OR Tonga[ot] OR Trinidad[ot] OR Tobago[ot] OR Tunisia[ot] OR Turkey[ot] OR Turkmenistan[ot] OR Turkmen[ot] OR Uganda[ot] OR Ukraine[ot] OR Uruguay[ot] OR USSR[ot] OR Soviet Union[ot] OR Union of Soviet Socialist Republics[ot] OR Uzbekistan[ot] OR Uzbek OR Vanuatu[ot] OR New Hebrides[ot] OR Venezuela[ot] OR Vietnam[ot] OR Viet Nam[ot] OR West Bank[ot] OR Yemen[ot] OR Yugoslavia[ot] OR Zambia[ot] OR Zimbabwe[ot] OR Rhodesia[ot]) OR(Developing Countries[Mesh:noexp] OR Africa[Mesh:noexp] OR Africa, Northern[Mesh:noexp] OR Africa South of the Sahara[Mesh:noexp] OR Africa, Central[Mesh:noexp] OR Africa, Eastern[Mesh:noexp] OR Africa, Southern[Mesh:noexp] OR Africa, Western[Mesh:noexp] OR Asia[Mesh:noexp] OR Asia, Central[Mesh:noexp] OR Asia, Southeastern[Mesh:noexp] OR Asia, Western[Mesh:noexp] OR Caribbean Region[Mesh:noexp] OR West Indies[Mesh:noexp] OR South America[Mesh:noexp] OR Latin America[Mesh:noexp] OR Central America[Mesh:noexp] OR Afghanistan[Mesh:noexp] OR Albania[Mesh:noexp] OR Algeria[Mesh:noexp] OR American Samoa[Mesh:noexp] OR Angola[Mesh:noexp] OR "Antigua and Barbuda"[Mesh:noexp] OR Argentina[Mesh:noexp] OR Armenia[Mesh:noexp] OR Azerbaijan[Mesh:noexp] OR Bahrain[Mesh:noexp] OR Bangladesh[Mesh:noexp] OR Barbados[Mesh:noexp] OR Benin[Mesh:noexp] OR Byelarus[Mesh:noexp] OR Belize[Mesh:noexp] OR Bhutan[Mesh:noexp] OR Bolivia[Mesh:noexp] OR Bosnia-Herzegovina[Mesh:noexp] OR Botswana[Mesh:noexp] OR Brazil[Mesh:noexp] OR Bulgaria[Mesh:noexp] OR Burkina Faso[Mesh:noexp] OR Burundi[Mesh:noexp] OR Cambodia[Mesh:noexp] OR Cameroon[Mesh:noexp] OR Cape Verde[Mesh:noexp] OR Central African Republic[Mesh:noexp] OR Chad[Mesh:noexp] OR Chile[Mesh:noexp] OR China[Mesh:noexp] OR Colombia[Mesh:noexp] OR Comoros[Mesh:noexp] OR Congo[Mesh:noexp] OR Costa Rica[Mesh:noexp] OR Cote d'Ivoire[Mesh:noexp] OR Croatia[Mesh:noexp] OR Cuba[Mesh:noexp] OR Cyprus[Mesh:noexp] OR Czechoslovakia[Mesh:noexp] OR Czech Republic[Mesh:noexp] OR Slovakia[Mesh:noexp] OR Djibouti[Mesh:noexp] OR "Democratic Republic of the Congo"[Mesh:noexp] OR Dominica[Mesh:noexp] OR Dominican Republic[Mesh:noexp] OR East Timor[Mesh:noexp] OR Ecuador[Mesh:noexp] OR Egypt[Mesh:noexp] OR El Salvador[Mesh:noexp] OR Eritrea[Mesh:noexp] OR Estonia[Mesh:noexp] OR Ethiopia[Mesh:noexp] OR Fiji[Mesh:noexp] OR Gabon[Mesh:noexp] OR Gambia[Mesh:noexp] OR "Georgia (Republic)"[Mesh:noexp] OR Ghana[Mesh:noexp] OR Greece[Mesh:noexp] OR Grenada[Mesh:noexp] OR Guatemala[Mesh:noexp] OR Guinea[Mesh:noexp] OR Guinea-Bissau[Mesh:noexp] OR Guam[Mesh:noexp] OR Guyana[Mesh:noexp] OR Haiti[Mesh:noexp] OR Honduras[Mesh:noexp] OR Hungary[Mesh:noexp] OR India[Mesh:noexp] OR Indonesia[Mesh:noexp] OR Iran[Mesh:noexp] OR Iraq[Mesh:noexp] OR Jamaica[Mesh:noexp] OR Jordan[Mesh:noexp] OR Kazakhstan[Mesh:noexp] OR Kenya[Mesh:noexp] OR Korea[Mesh:noexp] OR Kosovo[Mesh:noexp] OR Kyrgyzstan[Mesh:noexp] OR Laos[Mesh:noexp] OR Latvia[Mesh:noexp] OR Lebanon[Mesh:noexp] OR Lesotho[Mesh:noexp] OR Liberia[Mesh:noexp] OR Libya[Mesh:noexp] OR Lithuania[Mesh:noexp] OR Macedonia[Mesh:noexp] OR Madagascar[Mesh:noexp] OR Malaysia[Mesh:noexp] OR Malawi[Mesh:noexp] OR Mali[Mesh:noexp] OR Malta[Mesh:noexp] OR Mauritania[Mesh:noexp] OR Mauritius[Mesh:noexp] OR Mexico[Mesh:noexp] OR Micronesia[Mesh:noexp] OR Middle East[Mesh:noexp] OR Moldova[Mesh:noexp] OR Mongolia[Mesh:noexp] OR Montenegro[Mesh:noexp] OR Morocco[Mesh:noexp] OR Mozambique[Mesh:noexp] OR Myanmar[Mesh:noexp] OR Namibia[Mesh:noexp] OR Nepal[Mesh:noexp] OR Netherlands Antilles[Mesh:noexp] OR New Caledonia[Mesh:noexp] OR Nicaragua[Mesh:noexp] OR Niger[Mesh:noexp] OR Nigeria[Mesh:noexp] OR Oman[Mesh:noexp] OR Pakistan[Mesh:noexp] OR Palau[Mesh:noexp] OR Panama[Mesh:noexp] OR Papua New Guinea[Mesh:noexp] OR Paraguay[Mesh:noexp] OR Peru[Mesh:noexp] OR Philippines[Mesh:noexp] OR Poland[Mesh:noexp] OR Portugal[Mesh:noexp] OR Puerto Rico[Mesh:noexp] OR Romania[Mesh:noexp] OR Russia[Mesh:noexp] OR "Russia (Pre-1917)"[Mesh:noexp] OR Rwanda[Mesh:noexp] OR "Saint Kitts and Nevis"[Mesh:noexp] OR Saint Lucia[Mesh:noexp] OR "Saint Vincent and the Grenadines"[Mesh:noexp] OR Samoa[Mesh:noexp] OR Saudi Arabia[Mesh:noexp] OR Senegal[Mesh:noexp] OR Serbia[Mesh:noexp] OR Montenegro[Mesh:noexp] OR Seychelles[Mesh:noexp] OR Sierra Leone[Mesh:noexp] OR Slovenia[Mesh:noexp] OR Sri Lanka[Mesh:noexp] OR Somalia[Mesh:noexp] OR South Africa[Mesh:noexp] OR Sudan[Mesh:noexp] OR Suriname[Mesh:noexp] OR Swaziland[Mesh:noexp] OR Syria[Mesh:noexp] OR Tajikistan[Mesh:noexp] OR Tanzania[Mesh:noexp] OR Thailand[Mesh:noexp] OR Togo[Mesh:noexp] OR Tonga[Mesh:noexp] OR "Trinidad and Tobago"[Mesh:noexp] OR Tunisia[Mesh:noexp] OR Turkey[Mesh:noexp] OR Turkmenistan[Mesh:noexp] OR Uganda[Mesh:noexp] OR Ukraine[Mesh:noexp] OR Uruguay[Mesh:noexp] OR USSR[Mesh:noexp] OR Uzbekistan[Mesh:noexp] OR Vanuatu[Mesh:noexp] OR Venezuela[Mesh:noexp] OR Vietnam[Mesh:noexp] OR Yemen[Mesh:noexp] OR Yugoslavia[Mesh:noexp] OR Zambia[Mesh:noexp] OR Zimbabwe[Mesh:noexp]) | 1312 |
| Scopus | TITLE-ABS-KEY("health seeking" OR "health-seeking" OR "care seeking" OR "care-seeking" OR "healthcare seeking" OR "healthcare-seeking" OR "treatment seeking" OR "treatment-seeking" OR "health behaviour" OR "health behavior" OR "health care behaviour" OR "health care behavior" OR "healthcare behaviour" OR "healthcare behavior" OR "illness behaviour" OR "illness behavior" OR "health service utilisation" OR "health service utilization" OR "health care use" OR "healthcare use")  AND TITLE-ABS-KEY(fever* OR febrile OR hyperthermia OR pyrexi* OR malaria OR pneumonia)  AND TITLE-ABS-KEY(Infan* OR newborn* OR new-born* OR perinat* OR neonat* OR baby* OR babies OR toddler* OR minors* OR boy* OR girl* OR kid OR kids OR child* OR pediatrics OR pediatric* OR paediatric* OR peadiatric*)  AND TITLE-ABS-KEY((Africa or Asia or Caribbean or "West Indies" or "South America" or "Latin America" or "Central America") OR (Afghanistan or Albania or Algeria or Angola or Antigua or Barbuda or Argentina or Armenia or Armenian or Aruba or Azerbaijan or Bahrain or Bangladesh or Barbados or Benin or Byelarus or Byelorussian or Belarus or Belorussian or Belorussia or Belize or Bhutan or Bolivia or Bosnia or Herzegovina or Hercegovina or Botswana or Brasil or Brazil or Bulgaria or "Burkina Faso" or "Burkina Fasso" or "Upper Volta" or Burundi or Urundi or Cambodia or "Khmer Republic" or Kampuchea or Cameroon or Cameroons or Cameron or Camerons or "Cape Verde" or "Central African Republic" or Chad or Chile or China or Colombia or Comoros or "Comoro Islands" or Comores or Mayotte or Congo or Zaire or "Costa Rica" or "Cote d'Ivoire" or "Ivory Coast" or Croatia or Cuba or Cyprus or Czechoslovakia or "Czech Republic" or Slovakia or "Slovak Republic" or Djibouti or "French Somaliland" or Dominica or "Dominican Republic" or "East Timor" or "East Timur" or "Timor Leste" or Ecuador or Egypt or "United Arab Republic" or "El Salvador" or Eritrea or Estonia or Ethiopia or Fiji or Gabon or "Gabonese Republic" or Gambia or Gaza or "Georgia Republic" or "Georgian Republic" or Ghana or "Gold Coast" or Greece or Grenada or Guatemala or Guinea or Guam or Guiana or Guyana or Haiti or Honduras or Hungary or India or Maldives or Indonesia or Iran or Iraq or "Isle of Man" or Jamaica or Jordan or Kazakhstan or Kazakh or Kenya or Kiribati or Korea or Kosovo or Kyrgyzstan or Kirghizia or "Kyrgyz Republic" or Kirghiz or Kirgizstan or "Lao PDR" or Laos or Latvia or Lebanon or Lesotho or Basutoland or Liberia or Libya or Lithuania or Macedonia or Madagascar or "Malagasy Republic" or Malaysia or Malaya or Malay or Sabah or Sarawak or Malawi or Nyasaland or Mali or Malta or "Marshall Islands" or Mauritania or Mauritius or "Agalega Islands" or Mexico or Micronesia or "Middle East" or Moldova or Moldovia or Moldovian or Mongolia or Montenegro or Morocco or Ifni or Mozambique or Myanmar or Myanma or Burma or Namibia or Nepal or Netherlands Antilles or New Caledonia or Nicaragua or Niger or Nigeria or "Northern Mariana Islands" or Oman or Muscat or Pakistan or Palau or Palestine or Panama or Paraguay or Peru or Philippines or Philipines or Phillipines or Phillippines or Poland or Portugal or "Puerto Rico" or Romania or Rumania or Roumania or Russia or Russian or Rwanda or Ruanda or "Saint Kitts" or "St Kitts" or Nevis or "Saint Lucia" or "St Lucia" or "Saint Vincent" or "St Vincent" or Grenadines or Samoa or "Samoan Islands" or "Navigator Island" or "Navigator Islands" or "Sao Tome" or "Saudi Arabia" or Senegal or Serbia or Montenegro or Seychelles or "Sierra Leone" or Slovenia or "Sri Lanka" or Ceylon or "Solomon Islands" or Somalia or "South Africa" or Sudan or Suriname or Surinam or Swaziland or Syria or Tajikistan or Tadzhikistan or Tadjikistan or Tadzhik or Tanzania or Thailand or Togo or "Togolese Republic" or Tonga or Trinidad or Tobago or Tunisia or Turkey or Turkmenistan or Turkmen or Uganda or Ukraine or Uruguay or USSR or "Soviet Union" or "Union of Soviet Socialist Republics" or Uzbekistan or Uzbek or Vanuatu or "New Hebrides" or Venezuela or Vietnam or Viet Nam or "West Bank" or Yemen or Yugoslavia or Zambia or Zimbabwe or Rhodesia) OR ((developing or "less* developed" or "under developed" or underdeveloped or "middle income" or "low* income" or underserved or "under served" or deprived or poor*) pre/0 (countr* or nation? or population? or world)) OR ((developing or "less* developed" or "under developed" or underdeveloped or "middle income" or "low* income") pre/0 (economy or economies)) OR (low* pre/0 (gdp or gnp or "gross domestic" or "gross national")) OR (low pre/3 middle pre/3 countr*) OR (lmic or lmics or "third world" or "lami countr*") OR "transitional countr*") | 314 |
| WHOLIS | ("health seeking" OR "health-seeking" OR "care seeking" OR "care-seeking" OR "healthcare seeking" OR "healthcare-seeking" OR "treatment seeking" OR "treatment-seeking" OR "health behaviour" OR "health behavior" OR "health care behaviour" OR "health care behavior" OR "healthcare behaviour" OR "healthcare behavior" OR "illness behaviour" OR "illness behavior" OR "health service utilisation" OR "health service utilization" OR "health care use" OR "healthcare use")  AND (fever* OR febrile OR hyperthermia OR pyrexi* OR malaria OR pneumonia)  AND (Infan* OR newborn* OR new-born* OR perinat* OR neonat* OR baby* OR babies OR toddler* OR minors* OR boy* OR girl* OR kid OR kids OR child* OR pediatrics OR pediatric* OR paediatric* OR peadiatric*)  AND (((Africa or Asia or Caribbean or "West Indies" or "South America" or "Latin America" or "Central America") OR (Afghanistan or Albania or Algeria or Angola or Antigua or Barbuda or Argentina or Armenia or Armenian or Aruba or Azerbaijan or Bahrain or Bangladesh or Barbados or Benin or Byelarus or Byelorussian or Belarus or Belorussian or Belorussia or Belize or Bhutan or Bolivia or Bosnia or Herzegovina or Hercegovina or Botswana or Brasil or Brazil or Bulgaria or "Burkina Faso" or "Burkina Fasso" or "Upper Volta" or Burundi or Urundi or Cambodia or "Khmer Republic" or Kampuchea or Cameroon or Cameroons or Cameron or Camerons or "Cape Verde" or "Central African Republic" or Chad or Chile or China or Colombia or Comoros or "Comoro Islands" or Comores or Mayotte or Congo or Zaire or "Costa Rica" or "Cote d'Ivoire" or "Ivory Coast" or Croatia or Cuba or Cyprus or Czechoslovakia or "Czech Republic" or Slovakia or "Slovak Republic" or Djibouti or "French Somaliland" or Dominica or "Dominican Republic" or "East Timor" or "East Timur" or "Timor Leste" or Ecuador or Egypt or "United Arab Republic" or "El Salvador" or Eritrea or Estonia or Ethiopia or Fiji or Gabon or "Gabonese Republic" or Gambia or Gaza or "Georgia Republic" or "Georgian Republic" or Ghana or "Gold Coast" or Greece or Grenada or Guatemala or Guinea or Guam or Guiana or Guyana or Haiti or Honduras or Hungary or India or Maldives or Indonesia or Iran or Iraq or "Isle of Man" or Jamaica or Jordan or Kazakhstan or Kazakh or Kenya or Kiribati or Korea or Kosovo or Kyrgyzstan or Kirghizia or "Kyrgyz Republic" or Kirghiz or Kirgizstan or "Lao PDR" or Laos or Latvia or Lebanon or Lesotho or Basutoland or Liberia or Libya or Lithuania or Macedonia or Madagascar or "Malagasy Republic" or Malaysia or Malaya or Malay or Sabah or Sarawak or Malawi or Nyasaland or Mali or Malta or "Marshall Islands" or Mauritania or Mauritius or "Agalega Islands" or Mexico or Micronesia or "Middle East" or Moldova or Moldovia or Moldovian or Mongolia or Montenegro or Morocco or Ifni or Mozambique or Myanmar or Myanma or Burma or Namibia or Nepal or Netherlands Antilles or New Caledonia or Nicaragua or Niger or Nigeria or "Northern Mariana Islands" or Oman or Muscat or Pakistan or Palau or Palestine or Panama or Paraguay or Peru or Philippines or Philipines or Phillipines or Phillippines or Poland or Portugal or "Puerto Rico" or Romania or Rumania or Roumania or Russia or Russian or Rwanda or Ruanda or "Saint Kitts" or "St Kitts" or Nevis or "Saint Lucia" or "St Lucia" or "Saint Vincent" or "St Vincent" or Grenadines or Samoa or "Samoan Islands" or "Navigator Island" or "Navigator Islands" or "Sao Tome" or "Saudi Arabia" or Senegal or Serbia or Montenegro or Seychelles or "Sierra Leone" or Slovenia or "Sri Lanka" or Ceylon or "Solomon Islands" or Somalia or "South Africa" or Sudan or Suriname or Surinam or Swaziland or Syria or Tajikistan or Tadzhikistan or Tadjikistan or Tadzhik or Tanzania or Thailand or Togo or "Togolese Republic" or Tonga or Trinidad or Tobago or Tunisia or Turkey or Turkmenistan or Turkmen or Uganda or Ukraine or Uruguay or USSR or "Soviet Union" or "Union of Soviet Socialist Republics" or Uzbekistan or Uzbek or Vanuatu or "New Hebrides" or Venezuela or Vietnam or Viet Nam or "West Bank" or Yemen or Yugoslavia or Zambia or Zimbabwe or Rhodesia) OR ((developing or "less* developed" or "under developed" or underdeveloped or "middle income" or "low* income" or underserved or "under served" or deprived or poor*) pre/0 (countr* or nation? or population? or world)) OR ((developing or "less* developed" or "under developed" or underdeveloped or "middle income" or "low* income") pre/0 (economy or economies)) OR (low* pre/0 (gdp or gnp or "gross domestic" or "gross national")) OR (low pre/3 middle pre/3 countr*) OR (lmic or lmics or "third world" or "lami countr*") OR "transitional countr*") | 0 |

# Reference list for Table 3

Adedire EB, Asekun-Olarinmoye EO, Fawole O (2015) Maternal perception and care-seeking patterns for childhood febrile illnesses in rural communities of Osun State, south-western Nigeria. Sci J Public Health 2:636-643

Agyepong IA, Manderson L (1994) The diagnosis and management of fever at household level in the Greater Accra Region, Ghana. Acta Trop 58:317-330

Ahorlu CK, Koram KA, Ahorlu C et al. (2005) Community concepts of malaria-related illness with and without convulsions in southern Ghana. Malar J 4:47 doi:10.1186/1475-2875-4-47

Ajayi IO, Nsungwa-Sabiiti J, Siribie M et al. (2016) Feasibility of Malaria Diagnosis and Management in Burkina Faso, Nigeria, and Uganda: A Community-Based Observational Study. Clin Infect Dis 63:S245-S255 doi:10.1093/cid/ciw622

Akogun OB, Gundiri MA, Badaki JA et al. (2012) Febrile illness experience among Nigerian nomads. Int J Equity Health 11:5 doi:10.1186/1475-9276-11-5

Amuyunzu-Nyamongo M, Nyamongo IK (2006) Health Seeking Behaviour of Mothers of Under-Five-Year-Old Children in the Slum Communities of Nairobi, Kenya. Anthropol Med 13:25-40 doi:10.1080/13648470500516261

Anaba U, Hutchinson PL, Abegunde D et al. (2020) Pneumonia-related ideations, care-seeking, and treatment behaviors among children under 2 years with pneumonia symptoms in northwestern Nigeria. Pediatr Pulmonol 55 Suppl 1:S91-S103 doi:10.1002/ppul.24644

Awasthi S, Nichter M, Verma T et al. (2015) Revisiting community case management of childhood pneumonia: perceptions of caregivers and grass root health providers in Uttar Pradesh and Bihar, northern India. PLoS ONE 10:e0123135 doi:10.1371/journal.pone.0123135

Bantie GM, Meseret Z, Bedimo M et al. (2019) The prevalence and root causes of delay in seeking healthcare among mothers of under five children with pneumonia in hospitals of Bahir Dar city, North West Ethiopia. BMC Pediatr 19:482 doi:10.1186/s12887-019-1869-9

Baume C, Helitzer D, Kachur SP (2000) Patterns of care for childhood malaria in Zambia. Soc Sci Med 51:1491-1503

Beiersmann C, Sanou A, Wladarsch E et al. (2007) Malaria in rural Burkina Faso: local illness concepts, patterns of traditional treatment and influence on health-seeking behaviour. Malar J 6:106 doi:10.1186/1475-2875-6-106

Bruce N, Pope D, Arana B et al. (2014) Determinants of care seeking for children with pneumonia and diarrhea in Guatemala: implications for intervention strategies. Am J Public Health 104:647-657 doi:10.2105/ajph.2013.301658

Burton DC, Flannery B, Onyango B et al. (2011) Healthcare-seeking behaviour for common infectious disease-related illnesses in rural Kenya: a community-based house-to-house survey. J Health Popul Nutr 29:61-70

Chibwana AI, Mathanga DP, Chinkhumba J et al. (2009) Socio-cultural predictors of health-seeking behaviour for febrile under-five children in Mwanza-Neno district, Malawi. Malar J 8:219 doi:10.1186/1475-2875-8-219

Comoro C, Nsimba SE, Warsame M et al. (2003) Local understanding, perceptions and reported practices of mothers/guardians and health workers on childhood malaria in a Tanzanian district--implications for malaria control. Acta Trop 87:305-313 doi:10.1016/s0001-706x(03)00113-x

Dada OA, Omokhodion FO (2007) Home management of malaria by mothers of children under-five in Abeokuta, Southwest Nigeria. Trop Doct 37:217-219 doi:10.1258/004947507782332982

de Savigny D, Mayombana C, Mwageni E et al. (2004) Care-seeking patterns for fatal malaria in Tanzania. Malar J 3:27 doi:10.1186/1475-2875-3-27

Desmond NA, Nyirenda D, Dube Q et al. (2013) Recognising and treatment seeking for acute bacterial meningitis in adults and children in resource-poor settings: a qualitative study. PloS One 8:e68163 doi:10.1371/journal.pone.0068163

Deutscher M, Beneden CV, Burton D et al. (2012) Putting surveillance data into context: the role of health care utilization surveys in understanding population burden of pneumonia in developing countries. J Epidemiol Glob Health 2:73-81 doi:10.1016/j.jegh.2012.03.001

Dillip A, Alba S, Mshana C et al. (2012) Acceptability--a neglected dimension of access to health care: findings from a study on childhood convulsions in rural Tanzania. BMC Health Serv Res 12:113 doi:10.1186/1472-6963-12-113

Dillip A, Hetzel MW, Gosoniu D et al. (2009) Socio-cultural factors explaining timely and appropriate use of health facilities for degedege in south-eastern Tanzania. Malar J 8:144 doi:10.1186/1475-2875-8-144

Do M, Babalola S, Awantang G et al. (2018) Associations between malaria-related ideational factors and care-seeking behavior for fever among children under five in Mali, Nigeria, and Madagascar. PLoS ONE 13:e0191079 doi:10.1371/journal.pone.0191079

Druetz T, Ridde V, Kouanda S et al. (2015) Utilization of community health workers for malaria treatment: results from a three-year panel study in the districts of Kaya and Zorgho, Burkina Faso. Malar J 14:71 doi:10.1186/s12936-015-0591-9

Elimian KO, Myles PR, Phalkey R et al. (2020) 'Everybody in Nigeria is a doctor...': a qualitative study of stakeholder perspectives on lay diagnosis of malaria and pneumonia in Nigeria. J Public Health (Oxf) 42:353-361 doi:10.1093/pubmed/fdaa015

Ellis AA, Traore S, Doumbia S et al. (2012) Treatment actions and treatment failure: case studies in the response to severe childhood febrile illness in Mali. BMC Public Health 12:946 doi:10.1186/1471-2458-12-946

Escribano-Ferrer B, Gyapong M, Bruce J et al. (2017) Effectiveness of two community-based strategies on disease knowledge and health behaviour regarding malaria, diarrhoea and pneumonia in Ghana. BMC Public Health 17:948 doi:10.1186/s12889-017-4964-6

Ewing VL, Tolhurst R, Kapinda A et al. (2015) Understanding Interpretations of and Responses to Childhood Fever in the Chikhwawa District of Malawi. PLoS ONE 10:e0125439 doi:10.1371/journal.pone.0125439

Ferdous F, Ahmed S, Das SK et al. (2018) Pneumonia mortality and healthcare utilization in young children in rural Bangladesh: a prospective verbal autopsy study. Trop Med Health 46:17 doi:10.1186/s41182-018-0099-4

Ferdous F, Dil Farzana F, Ahmed S et al. (2014) Mothers' perception and healthcare seeking behavior of pneumonia children in rural Bangladesh. ISRN Family Med 2014:690315 doi:10.1155/2014/690315

Foster D, Vilendrer S (2009) Two treatments, one disease: childhood malaria management in Tanga, Tanzania. Malar J 8:240 doi:10.1186/1475-2875-8-240

Hausmann-Muela S (2000) Community understanding of malaria, and treatment seeking behaviour, in a holoendemic area of southeastern Tanzania. PhD thesis, University of Basel

Hildenwall H, Lindkvist J, Tumwine JK et al. (2009a) Low validity of caretakers' reports on use of selected antimalarials and antibiotics in children with severe pneumonia at an urban hospital in Uganda. Trans R Soc Trop Med Hyg 103:95-101 doi:10.1016/j.trstmh.2008.04.046

Hildenwall H, Nantanda R, Tumwine JK et al. (2009b) Care-seeking in the development of severe community acquired pneumonia in Ugandan children. Ann Trop Paediatr 29:281-289 doi:10.1179/027249309X12547917869005

Hildenwall H, Tomson G, Kaija J et al. (2008) "I never had the money for blood testing" - caretakers' experiences of care-seeking for fatal childhood fevers in rural Uganda - a mixed methods study. BMC Int Health Hum Rights 8:12 doi:10.1186/1472-698X-8-12

Houéto D, d'Hoore W, Ouendo EM et al. (2007) [Childhood fever in a zone of malaria prevalence in Benin: qualitative analysis of factors linked to seeking care]. Sante Publique 19:363-372

Kaatano GM, Muro AIS, Medard M (2006) Caretaker's perceptions, attitudes and practices regarding childhood febrile illness and diarrhoeal diseases among riparian communities of Lake Victoria, Tanzania. Tanzan Health Res Bull 8:155-161

Källander K, Hildenwall H, Waiswa P et al. (2008) Delayed care seeking for fatal pneumonia in children aged under five years in Uganda: a case-series study. Bull World Health Organ 86:332-338

Kamat VR (2006) "I thought it was only ordinary fever!" cultural knowledge and the micropolitics of therapy seeking for childhood febrile illness in Tanzania. Soc Sci Med 62:2945-2959 doi:10.1016/j.socscimed.2005.11.042

Kamat VR (2008) Dying under the bird's shadow: narrative representations of degedege and child survival among the Zaramo of Tanzania. Med Anthropol Q 22:67-93 doi:10.1111/j.1548-1387.2008.00004.x

Kaona FAD, Tuba M (2005) A qualitative study to identify community structures for management of severe malaria: a basis for introducing rectal artesunate in the under five years children in Nakonde District of Zambia. BMC Public Health 5:28 doi:10.1186/1471-2458-5-28

Kapoor SK, Reddaiah VP, Murthy GV (1990) Knowledge, attitude and practices regarding acute respiratory infections. Indian J Pediatr 57:533-535 doi:10.1007/bf02726763

Kassam R, Sekiwunga R, Collins JB et al. (2016a) Caregivers' treatment-seeking behaviors and predictors of whether a child received an appropriate antimalarial treatment: a household survey in rural Uganda. BMC Infect Dis 16:478 doi:10.1186/s12879-016-1815-5

Kassam R, Sekiwunga R, MacLeod D et al. (2016b) Patterns of treatment-seeking behaviors among caregivers of febrile young children: a Ugandan multiple case study. BMC Public Health 16:160 doi:10.1186/s12889-016-2813-7

Kassile T, Lokina R, Mujinja P et al. (2014) Determinants of delay in care seeking among children under five with fever in Dodoma region, central Tanzania: a cross-sectional study. Malar J 13:348 doi:10.1186/1475-2875-13-348

Kerai S, Nisar I, Muhammad I et al. (2019) A Community-Based Survey on Health-Care Utilization for Pneumonia in Children in Peri-Urban Slums of Karachi, Pakistan. Am J Trop Med Hyg 101:1034-1041 doi:10.4269/ajtmh.18-0656

Kosai H, Tamaki R, Saito M et al. (2015) Incidence and Risk Factors of Childhood Pneumonia-Like Episodes in Biliran Island, Philippines--A Community-Based Study. PLoS ONE 10:e0125009 doi:10.1371/journal.pone.0125009

Lindblade KA, O'Neill DB, Mathanga DP et al. (2000) Treatment for clinical malaria is sought promptly during an epidemic in a highland region of Uganda. Trop Med Int Health 5:865-875

Luque JS, Whiteford LM, Tobin GA (2008) Maternal recognition and health care-seeking behavior for acute respiratory infection in children in a rural Ecuadorian county. Matern Child Health J 12:287-297 doi:10.1007/s10995-007-0249-5

Makundi EA, Malebo HM, Mhame P et al. (2006) Role of traditional healers in the management of severe malaria among children below five years of age: the case of Kilosa and Handeni Districts, Tanzania. Malar J 5:58 doi:10.1186/1475-2875-5-58

Malik EM, Hanafi K, Ali SH et al. (2006) Treatment-seeking behaviour for malaria in children under five years of age: implication for home management in rural areas with high seasonal transmission in Sudan. Malar J 5:60 doi:10.1186/1475-2875-5-60

Mayombana CC (2004) Local understanding of practices related to IMCI interventions in Eastern Tanzania. PhD thesis, University of Basel

McNee A, Khan N, Dawson S et al. (1995) Responding to cough: Boholano illness classification and resort to care in response to childhood ARI. Soc Sci Med 40:1279-1289 doi:10.1016/0277-9536(94)00242-l

Mitiku I, Assefa A (2017) Caregivers' perception of malaria and treatment-seeking behaviour for under five children in Mandura District, West Ethiopia: a cross-sectional study. Malar J 16:144 doi:10.1186/s12936-017-1798-8

Müller O, Traoré C, Becher H et al. (2003) Malaria morbidity, treatment-seeking behaviour, and mortality in a cohort of young children in rural Burkina Faso. Trop Med Int Health 8:290-296

Munthali AC (2003) Perceptions about the aetilogy, treatment and prevention of convulsions in under-five children in Rumphi. Malawi Med J 15:11-12 doi:10.4314/mmj.v15i1.10771

Munthali AC (2005) Managing Malaria in Under-Five Children in a Rural Malawian Village. Nord J Afr Stud 14:127-146

Muro F, Meta J, Renju J et al. (2017) "It is good to take her early to the doctor" - mothers' understanding of childhood pneumonia symptoms and health care seeking in Kilimanjaro region, Tanzania. BMC Int Health Hum Rights 17:27 doi:10.1186/s12914-017-0135-1

Naheed A, Breiman RF, Islam MS et al. (2019) Disparities by sex in care-seeking behaviors and treatment outcomes for pneumonia among children admitted to hospitals in Bangladesh. PLoS ONE 14:e0213238 doi:10.1371/journal.pone.0213238

Najnin N, Bennett CM, Luby SP (2011) Inequalities in care-seeking for febrile illness of under-five children in urban Dhaka, Bangladesh. J Health Popul Nutr 29:523-531

Nsungwa-Sabiiti J, Källander K, Nsabagasani X et al. (2004) Local fever illness classifications: implications for home management of malaria strategies. Trop Med Int Health 9:1191-1199 doi:10.1111/j.1365-3156.2004.01319.x

Okeke TA (2010) Improving malaria recognition, treatment and referral practices by training caretakers in rural Nigeria. J Biosoc Sci 42:325-339 doi:10.1017/S0021932009990484

Okeke TA, Okeibunor JC (2010) Rural-urban differences in health-seeking for the treatment of childhood malaria in south-east Nigeria. Health Policy 95:62-68 doi:10.1016/j.healthpol.2009.11.005

Oluchi SE, Manaf RA, Ismail S et al. (2019) Predictors of Health-Seeking Behavior for Fever Cases among Caregivers of Under-Five Children in Malaria-Endemic Area of Imo State, Nigeria. Int J Environ Res Public Health 16 doi:10.3390/ijerph16193752

Onyango D, Kikuvi G, Amukoye E et al. (2012) Risk factors of severe pneumonia among children aged 2-59 months in western Kenya: a case control study. Pan Afr Med J 13:45

Rashid SF, Hadi A, Afsana K et al. (2001) Acute respiratory infections in rural Bangladsh: cultural understandings, practices and the role of mothers and community health volunteers. Trop Med Int Health 6:249-255

Salah MT, Adam I, Malik EM (2007) Care-seeking behavior for Fever in children under five years in an urban area in eastern Sudan. J Family Community Med 14:25-28

Sankarapandian V, Friberg IK, John SM et al. (2011) Reported healthcare utilisation for childhood respiratory illnesses in Vellore, South India. Int Health 3:199-205 doi:10.1016/j.inhe.2011.04.001

Simba DO, Kakoko DC, Warsame M et al. (2010) Understanding caretakers' dilemma in deciding whether or not to adhere with referral advice after pre-referral treatment with rectal artesunate. Malar J 9:123 doi:10.1186/1475-2875-9-123

Simba DO, Warsame M, Kimbute O et al. (2009) Factors influencing adherence to referral advice following pre-referral treatment with artesunate suppositories in children in rural Tanzania. Trop Med Int Health 14:775-783 doi:10.1111/j.1365-3156.2009.02299.x

Siribié M, Ajayi IO, Nsungwa-Sabiiti J et al. (2016) Compliance With Referral Advice After Treatment With Prereferral Rectal Artesunate: A Study in 3 Sub-Saharan African Countries. Clin Infect Dis 63:S283-S289 doi:10.1093/cid/ciw627

Snavely ME, Maze MJ, Muiruri C et al. (2018) Sociocultural and health system factors associated with mortality among febrile inpatients in Tanzania: a prospective social biopsy cohort study. BMJ Glob Health 3:e000507 doi:10.1136/bmjgh-2017-000507

Straus L, Munguambe K, Bassat Q et al. (2011) Inherent illnesses and attacks: an ethnographic study of interpretations of childhood Acute Respiratory Infections (ARIs) in Manhica, southern Mozambique. BMC Public Health 11:556 doi:10.1186/1471-2458-11-556

Taffa N, Chepngeno G (2005) Determinants of health care seeking for childhood illnesses in Nairobi slums. Trop Med Int Health 10:240-245 doi:10.1111/j.1365-3156.2004.01381.x

Tarimo DS, Lwihula GK, Minjas JN et al. (2000) Mothers' perceptions and knowledge on childhood malaria in the holendemic Kibaha district, Tanzania: implications for malaria control and the IMCI strategy. Trop Med Int Health 5:179-184

Thomson A, Khogali M, de Smet M et al. (2011) Low referral completion of rapid diagnostic test-negative patients in community-based treatment of malaria in Sierra Leone. Malar J 10:94 doi:10.1186/1475-2875-10-94

Tinuade O, Iyabo R-A, Durotoye O (2010) Health-care-seeking behaviour for childhood illnesses in a resource-poor setting. J Paediatr Child Health 46:238-242 doi:10.1111/j.1440-1754.2009.01677.x

Tsukahara T, Ogura S, Sugahara T et al. (2015) The Choice of Healthcare Providers for Febrile Children after Introducing Non-professional Health Workers in a Malaria Endemic Area in Papua New Guinea. Front Public Health 3:275 doi:10.3389/fpubh.2015.00275

Ustrup M, Ngwira B, Stockman LJ et al. (2014) Potential barriers to healthcare in Malawi for under-five children with cough and fever: a national household survey. J Health Popul Nutr 32:68-78

Vaahtera M, Kulmala T, Maleta K et al. (2000) Epidemiology and predictors of infant morbidity in rural Malawi. Paediatr Perinat Epidemiol 14:363-371

Vermeersch A, Libaud-Moal A, Rodrigues A et al. (2014) Introducing the concept of a new pre-referral treatment for severely ill febrile children at community level: a sociological approach in Guinea-Bissau. Malar J 13:50 doi:10.1186/1475-2875-13-50

Warsame M, Kimbute O, Machinda Z et al. (2007) Recognition, perceptions and treatment practices for severe malaria in rural Tanzania: implications for accessing rectal artesunate as a pre-referral. PLoS ONE 2:e149 doi:10.1371/journal.pone.0000149

Yadav SP (2010) A study of treatment seeking behaviour for malaria and its management in febrile children in rural part of desert, Rajasthan, India. J Vector Borne Dis 47:235-242
